# Large‐Scale metabolomics: Predicting biological age using 10,133 routine untargeted LC–MS measurements

**DOI:** 10.1111/acel.13813

**Published:** 2023-03-19

**Authors:** Johan K. Lassen, Tingting Wang, Kirstine L. Nielsen, Jørgen B. Hasselstrøm, Mogens Johannsen, Palle Villesen

**Affiliations:** ^1^ Bioinformatics Research Center Aarhus University Aarhus Denmark; ^2^ Department of Forensic Medicine Aarhus University Aarhus Denmark; ^3^ Department of Clinical Medicine Aarhus University Aarhus Denmark

**Keywords:** accelerated aging, big data, inflammaging, machine learning, metabolomics, molecular biology of aging, tryptophan metabolism

## Abstract

Untargeted metabolomics is the study of all detectable small molecules, and in geroscience, metabolomics has shown great potential to describe the biological age—a complex trait impacted by many factors. Unfortunately, the sample sizes are often insufficient to achieve sufficient power and minimize potential biases caused by, for example, demographic factors. In this study, we present the analysis of biological age in ~10,000 toxicologic routine blood measurements. The untargeted screening samples obtained from ultra‐high pressure liquid chromatography‐quadruple time of flight mass spectrometry (UHPLC‐ QTOF) cover + 300 batches and + 30 months, lack pooled quality controls, lack controlled sample collection, and has previously only been used in small‐scale studies. To overcome experimental effects, we developed and tested a custom neural network model and compared it with existing prediction methods. Overall, the neural network was able to predict the chronological age with an rmse of 5.88 years (*r* ^*2*^ = 0.63) improving upon the 6.15 years achieved by existing normalization methods. We used the feature importance algorithm, Shapley Additive exPlanations (SHAP), to identify compounds related to the biological age. Most importantly, the model returned known aging markers such as kynurenine, indole‐3‐aldehyde, and acylcarnitines along with a potential novel aging marker, cyclo (leu‐pro). Our results validate the association of tryptophan and acylcarnitine metabolism to aging in a highly uncontrolled large‐s cale sample. Also, we have shown that by using robust computational methods it is possible to deploy large LC‐MS datasets for metabolomics studies to reduce the risk of bias and empower aging studies.

## Graphical abstract

We have used a highly uncontrolled cohort to validate some of the important pathways in aging and shown that routine untargeted measurements are a valuable resource in population‐wide studies of metabolomic biomarkers. The feature selection returned known biomarkers that strongly associate with inflammaging, the link between inflammation and aging. We also identified a new metabolite, cyclo (leu‐pro) which is not endogenous but plays antimicrobial and antiviral roles.

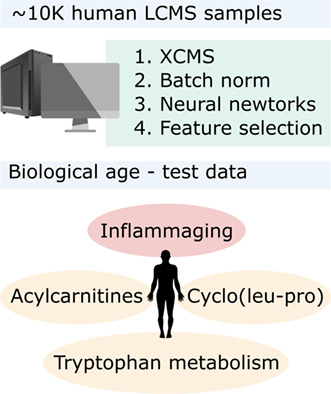

## 1 INTRODUCTION

Metabolomics is the study of small molecules in biological samples and is a strong tool to explain complex traits such as biological age—a product of lifestyle, genomic alterations, inflammation, time since birth, etc. (Franceschi et al. 2018; Kennedy et al. 2014). Several studies have modeled age and identified and validated aging markers, using targeted (known molecules) and untargeted (mostly unknown molecules) metabolomics (Ahadi et al. 2020; Auro et al., 2014; Darst et al. 2019; Rist et al., 2017; Robinson et al., 2020; Verri Hernandes et al., 2022; Yu et al., 2012). The output from the aging models is interpreted as biological age, and any discrepancy to the chronological age is interpreted as accelerated/decelerated aging which has been found to correlate with life expectancy (Hertel et al., 2016).

Human‐based large‐scale metabolomics studies of age exist, but most are focused on targeted metabolomics, that is, a small subset of compounds, because untargeted data are susceptible to experimental variance and computationally heavy to preprocess (T. Kim et al., 2021). Consequently, a great fraction of metabolites is neglected, although their inclusion might provide a better understanding of aging. To our knowledge the largest untargeted study is by O. Robinson et al. (2020) who successfully analyzed 2239 samples and predicted chronological age with high accuracy (Robinson et al., 2020). Despite this, factors such as demography introduce a risk of sampling bias, and we believe methods facilitating large‐scale untargeted metabolomics studies will help overcome these issues.

**FIGURE 1:**
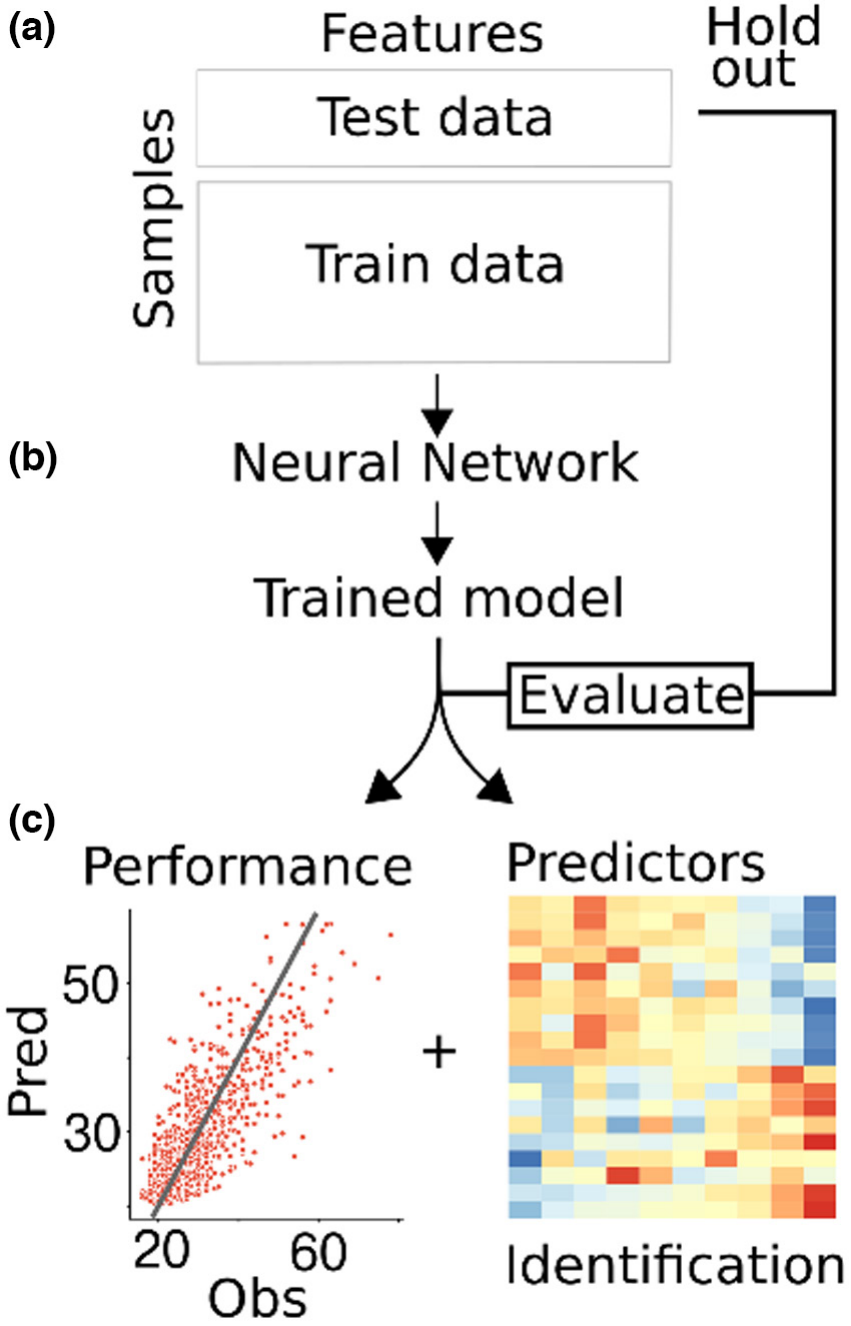
Statistical workflow. (a) The data are split into train and test partitions. (b) The training data are used to fit a neural network. (c) The trained model is evaluated using the test data, and the important features are extracted and identified.

In this study, we first introduce the use of untargeted mass spectrometry screening data (UHPLC‐QTOF in positive ESI mode) as a large‐scale resource of metabolomics data. Our data are acquired for toxicological screenings in routine forensic casework across several years and hundreds of analytical batches and we aim to deploy deploy it as a valuable resource in biological research. Second, we aim to predict the biological age of the donors to identify the potential biomarkers, and to validate our method against the existing literature (Figure 1).

## 2 RESULTS

### 2.1 Data

**FIGURE 2:**
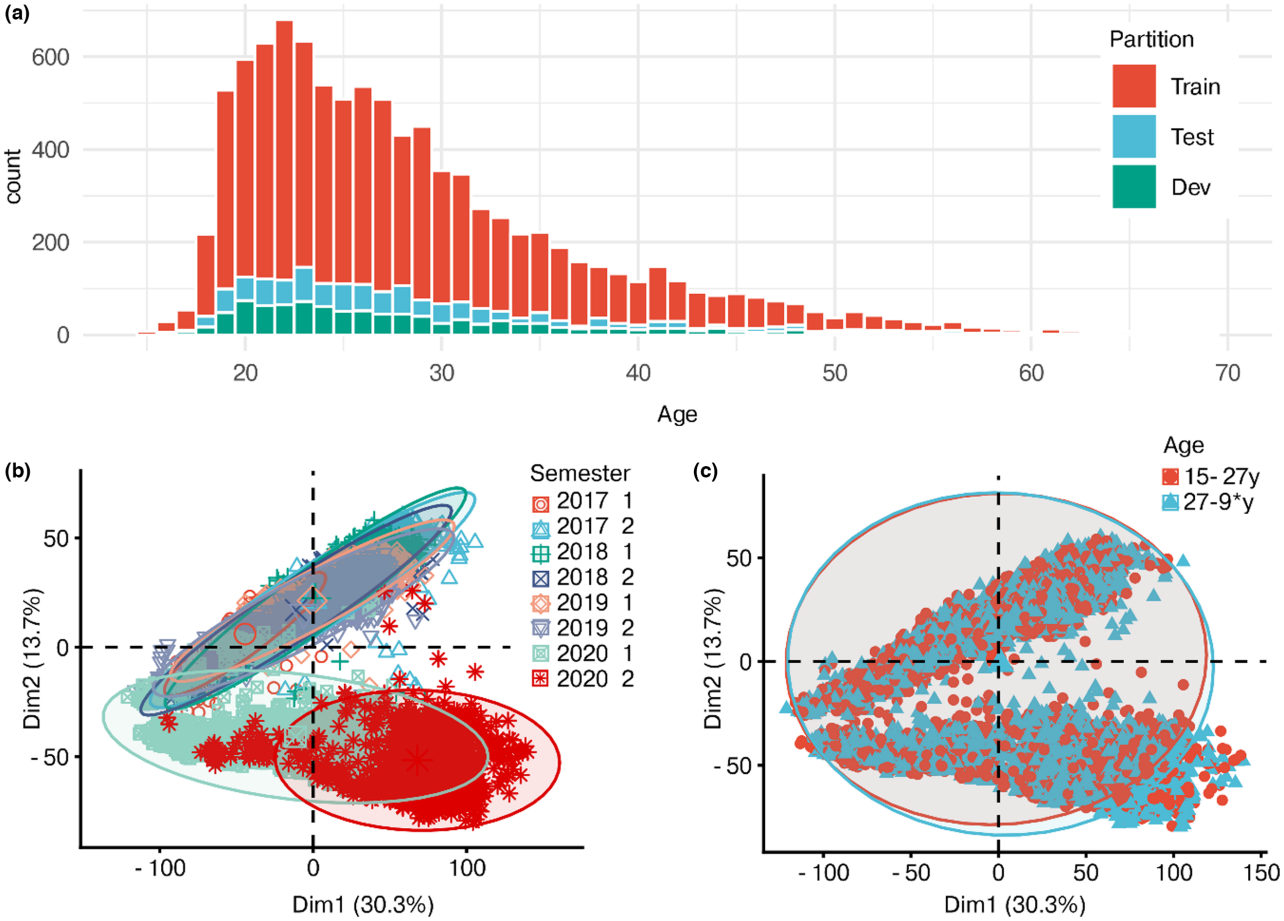
Raw data. (a) Distribution of age across all 10 k individuals, minimum 5 individuals per age, 13 individuals older than 75y. (b) Principal components of 4th root transformed data, colored by semester. (c) PCA colored by whether samples are more or less than the median age. The first 18 components colored by age are shown in Figure S2.

The data are antemortem whole blood samples (UHPLC‐QTOF) from drivers suspected of driving under the influence of drugs. The sampling period spans from January 2017 to December 2020 and contain 394 batches. The samples were kept at cooled conditions until reaching the analysis site typically within 2–7 days (see *experimental procedures*). 93% of the samples from the period are males with a mean age of 28.9 ± 9.2 SD. The age distribution is skewed, proposing a challenge for making balanced predictions in the machine learning models (Figure 2a). To comply with GDPR, all visualizations in this study are anonymized such that all age groups have +5 observations while calculations are based on all observations spanning from 15 to +90 years old.

XCMS peak calling yielded 12,686 features but features having >20% batch‐wise missing values were removed. Also, we removed female samples to avoid confounders and finally removed sample outliers using principal component analysis (PCA) (Rist et al., 2017). The final dataset used for the analysis contained 9929 samples and 8038 features. The observations were partitioned into training data (*n* = 7943) for fitting the models, development data (*n* = 993) for tuning NN hyperparameters, and testing data (*n* = 993) for hold‐out data model evaluation.

Strong experimental effects appeared on the PCA plot of the data (Figure 2b). Samples from 2020 clustered, as well as the semesters of 2020, which might be caused by high retention time drift (Figure S1). In sum, the first two components of the PCA accounted for 44% of the total variance in the data. The PCA did not reveal any patterns of age—neither in the first two components or in the following 16—indicating that only weak/few signals might explain age (Figure 2c, Figure S2). As the data lacked pooled quality controls (QCs) and had only a few internal standards, a QC‐based correction of the 394 batches was impossible.

### 2.2 Neural networks outperform existing normalization methods

We performed a small‐scale comparison of normalization methods suited for data lacking QCs (Table 1), including WaveICA2.0, Combat, quantile normalization, and row normalization (Deng et al., 2021; W. E. Johnson et al., 2007). We predicted age using machine learning and used the root mean squared error (RMSE) to assess the normalization methods. No method improved compared to naive methods such as row normalization, which achieved a test RMSE of 6.15 years. The first two components (PCA) were plotted for each normalized dataset to assess whether the batch effect was removed (Figure S3). WaveICA2.0 and Combat seemed to remove the batch structure but did not improve the machine learning performance.

**TABLE 1:** Model performance (RMSE) on test data from different normalization methods.

Preprocessing	Linear model	Elastic net	PLS	Random Forest	Gradient boosting	Neural network
Quantile	18.1	6.22	8.45	7.64	6.76	NA
Row Normalization	18.7	6.15	8.24	7.58	6.69	NA
WaveICA2.0	18.9	6.17	7.83	7.54	6.63	NA
Combat	19.2	6.21	7.61	7.53	6.57	NA
No normalization	18.6	6.23	8.63	7.63	6.77	5.88 (best)

The used preprocessing methods vary from naive normalizations (row normalization, quantile) to between‐batch normalization (Combat) and finally to between‐ *and* within‐batch normalization (WaveICA2.0). We assessed the effect size of the differences between model‐normalization pairs by bootstrapping the RMSE values and concluded the model type was more influential than the normalization type (Figure S4). Expanding with additional normalization methods would most likely not benefit the analysis and be outside the scope of this study.

As no normalization method improved the model RMSEs, we implemented a neural network (NN) as it offers a flexible framework for custom architectures. Consequently, we implemented a ratio layer in the NN motivated by the hypothesis that compound ratios have normalizing properties—that is, the ratio of two compounds is robust to general effects that dilute/enrich both compounds by the same factor in each sample. To avoid an explosion of model parameters leading to overfitting, the first layer of the NN consisted of only 12 nodes condensing the information of the 8038 features. The second layer contained all unique ratios of the first layer's output (1st layer outputs = 12 weights, ratios = 66), followed by two hidden layers and one output layer. See *experimental procedures* for a detailed motivation and the specific architecture.

As neural networks are heuristic methods, their fits differ from run to run despite using the same data and the same architecture. To ensure repeatability, we made a hyperparameter optimization to decide the optimal NN architecture and refitted the best architecture 1000 times on the training and development data. Finally, we evaluated the 1000 fitted NN models on the held‐out test data to get 1000 predictions per observation, which was averaged to one prediction. The testing data were not used for any model optimization or decision‐making in the process. We did not test any of the normalization methods on the NN model, as the architecture should automatically normalize, and the normalization methods had no or small effect on the RMSE of the simple models (Table 1).

**FIGURE 3:**
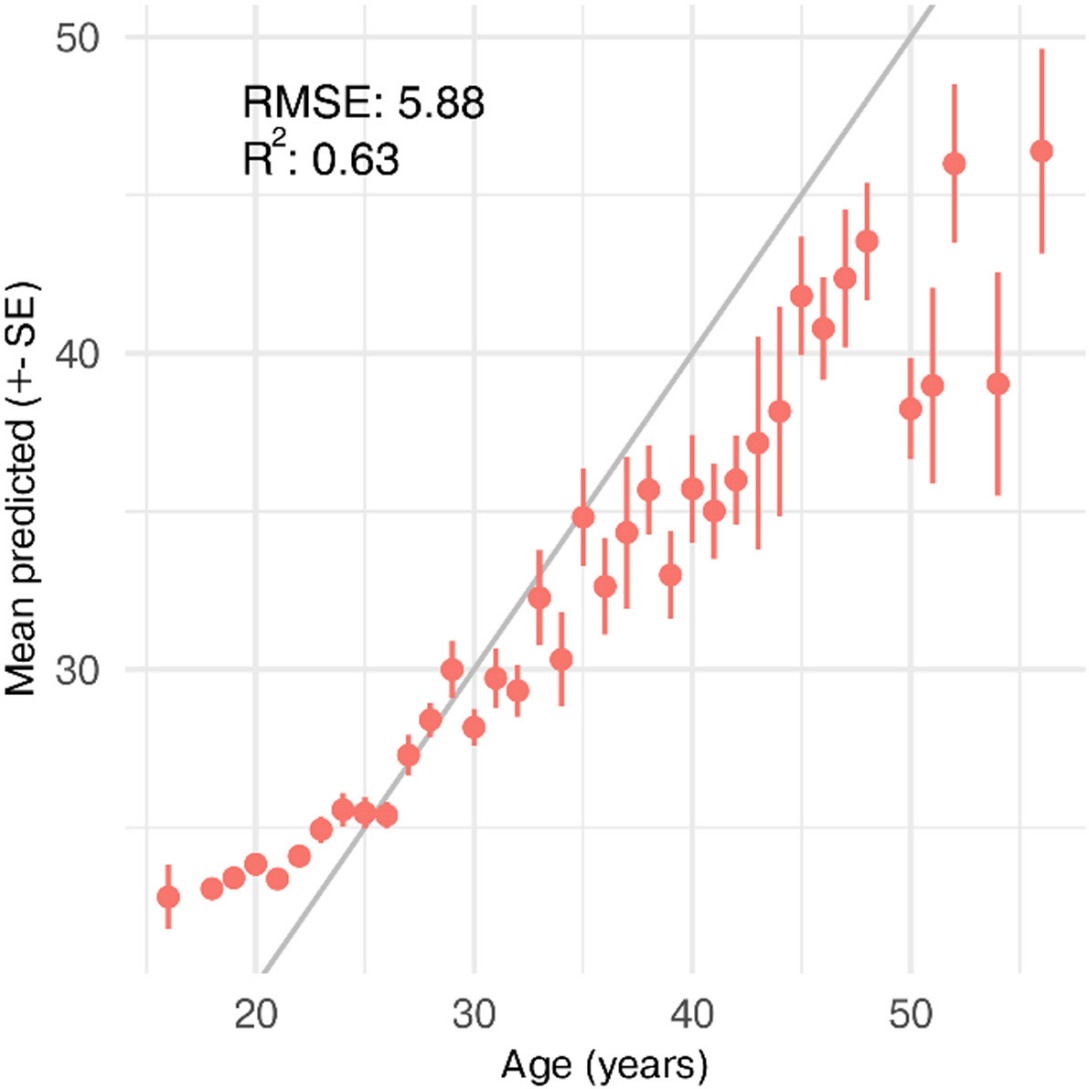
Performance of the averaged NN model. Predictions plotted against the observed age of the test data (*n* = 993). Mean and standard error is based on one prediction per observation (not repeated predictions). The grey line depicts the trend that perfect predictions should follow.

The averaged NN model predictions improved upon the normalization methods by achieving a test RMSE of 5.88 years (Table 1, Figure 3). Interestingly, the model predictions showed a smaller bias than the models from the model screening (Table 1) which underestimated the age of old individuals (Figure S5). Despite this, old samples were still underestimated in the model predictions, which potentially is caused by two reasons: First, the age distribution is skewed, making the model focus on the densest part of the distribution (21–29 years old). Second, the model might be mean biased, thus finding a balance of using the mean age (safe guess, never completely wrong) and the actual aging patterns. In practice, we must estimate accelerated aging by comparing individuals against the mean prediction of their age group and not the chronological age—otherwise, all 40‐year‐olds would appear 5 years younger.

### 2.3 SHapley additive exPlanation (SHAP) values identifies age associated features

**FIGURE 4:**
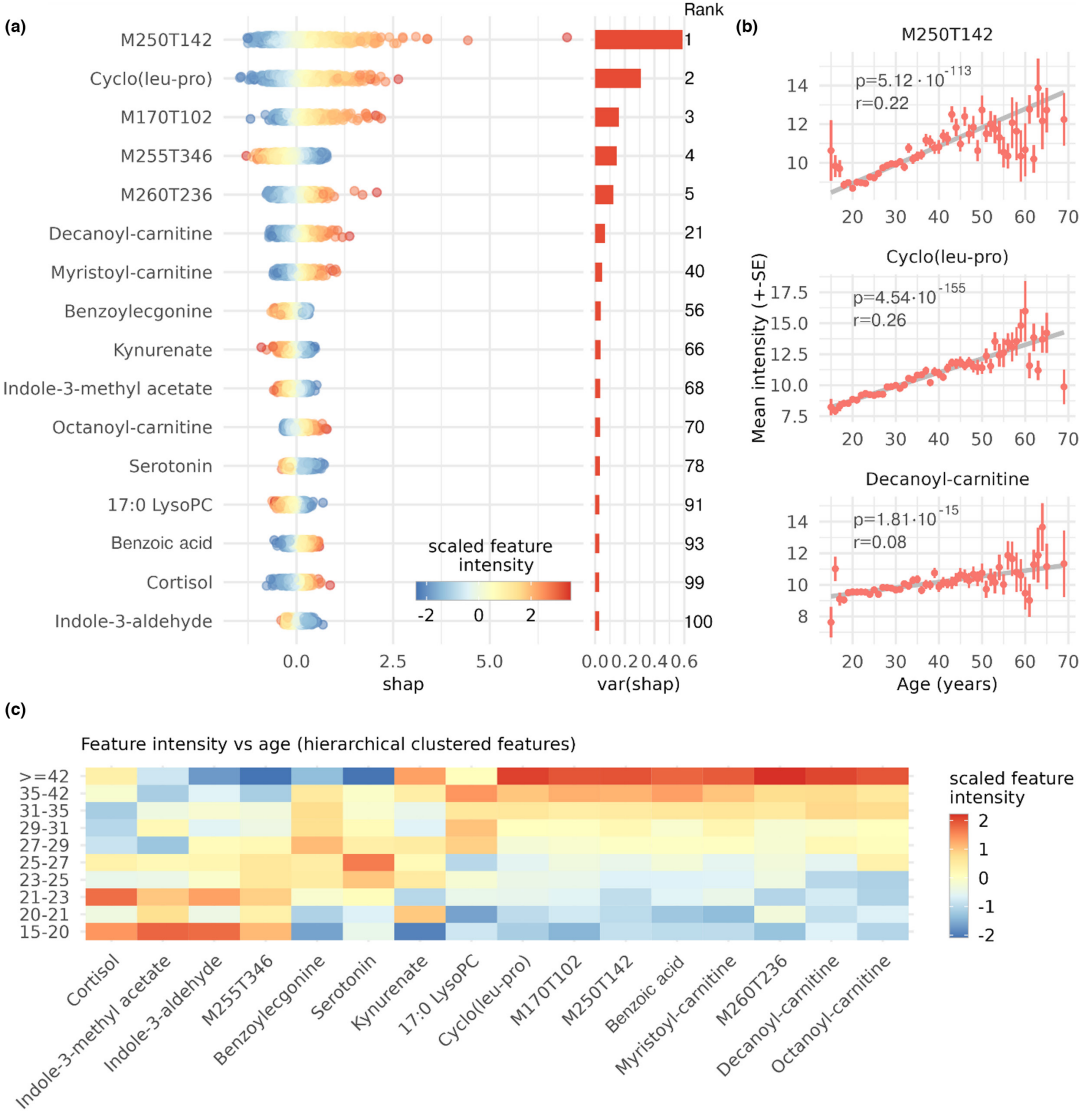
Feature selection by SHAP. (a) SHAP values (*n* = 993, test set only) of the top 5 features followed by all annotated features in top 100. One point represents one sample, all samples are represented in each feature, positive SHAP means older predictions, negative SHAP means younger predictions, and the color represents the scaled feature intensity. (b) Spearman correlations between the most important feature and age followed by the two most important annotations. Only ages with +5 observations shown. All data partitions used. (c) Mean intensity of each age decile scaled to z‐scores of all features from (a). Hierarchical clustered features and all data partitions used.

We extracted feature importance from each of the 1000 NN models using state‐of‐the‐art SHAP values that provide local importance and are computed by the model agnostic algorithm, SHAP (Lundberg & Lee, 2017). Local importance quantifies how much one feature affects one sample—for instance a feature's SHAP value is *positive* if the feature's intensity affects the sample prediction to be *older* than the mean age of the dataset. Features with SHAP values close to zero have small impact, meaning the SHAP variance reflects global (traditional) feature importance. The model agnosticism allows us to directly compare and average SHAP values between the 1000 fitted NN models that we also used to make the averaged NN predictions (Figure 4).

The calculated SHAP values indicated that the NN model primarily interpreted linear responses from the feature intensities. When the color gradient of a feature goes from blue to red (low to high feature intensity) the NN model interprets the feature as positively correlated with age (Figure 4a). On the contrary, red to blue gradients mean the model interprets features as negatively correlated with age. Hence, SHAP values provide a deeper insight into the models than the global importance in e.g., PLS and random forest.

Molecules positively correlated to the SHAP values included cyclo (leu‐pro), acylcarnitines, cortisol, and benzoic acid. Negatively SHAP correlated molecules included 17:0 Lyso PC, and the tryptophan pathway metabolites; serotonin, indole‐3‐aldehyde (I3A), kynurenate, and indole‐3‐methyl acetate. Some of the most important predictors remained unknown due to low fragmentation intensities or no database matches (see Data S1 for RT and m/z and methods for ID lvl 2 annotations).

Because of the ratios, the NN model potentially assigns high importance to normalizing features not correlated to age. Thus, we plotted the intensities of the most important predictors versus the age of the observations (Figure 4b and Figure 4c) and found strong positive Spearman correlations for M250T142, cyclo (leu‐pro), and decanoyl‐carnitine (Figure 4b). Also, the top 100 annotated features included increasing acylcarnitines and increasing/decreasing tryptophan derived metabolites (Figure 4c). Despite strong correlations, none of the compounds were useful predictors by themselves, as noise results in high standard deviations in each age group (Figure S6).

The NN model also interpreted two features inversely compared to the raw intensity vs. age. For cortisol which decreased with age (Figure 4c), the model associated high intensities with old age (Figure 4a), and for 17:0 LysoPC which increased with age (Figure 4c) the model associated low intensities with young age (Figure 4a). These inverse relationships might be caused by interactions or normalizing effects, but we can only hypothesize about this. For now, we refrain from investigating it further and omit these potential markers from the discussion due to the uncertainty about their effects in the data.

To further investigate the acylcarnitine and tryptophan pathways, we calculated false discovery rates based on Spearman p‐values using the correlations from all 8038 features vs. age (Figure S7). This analysis yielded 1867 correlated features at fdr < = 0.01. To quickly assess the combined information in the 1867 correlated features we performed a PCA and plotted the first 18 axes, but no age pattern appeared (Figure S8). Hence, we find it advantageous to use the non‐redundant, non‐covaried, and dense result from the NN model. However, the use of univariate analysis may supplement the NN model and elucidate some extra molecules of the pathways in action. Data S1 contains all presented statistics about the features.

### 2.4 Cyclo (leu‐pro)—a potential novel marker of age

Cyclo (leu‐pro) was the second most important metabolite in the NN model and had the smallest fdr value with a positive correlation to age of *r* = 0.26 (Spearman rho). The correlation was consistent throughout life, although additional observations will increase certainty for the +50y group (Figure 4b). Cyclo (leu‐pro) is a cyclic dipeptide (2,5‐diketopiperizene) found in diet, and is biologically active by its antimicrobial, antiviral, and antitumor properties (Rhee, 2004; Zhao et al., 2021).

### 2.5 Tryptophan metabolism

We also identified several tryptophan derivatives including kynurenate, serotonin, and the intestinal microorganism derived indole‐3‐aldehyde and indole‐3‐methyl acetate (Laurans et al., 2018; Pavlova et al., 2017). Both serotonin and the indoles were negatively correlated with age, while kynurenate did not show any strong association (Figure 4c). However, when investigating the univariate Spearman correlations to age, kynurenine—the linking metabolite between tryptophan and kynurenate—was positively correlated with age (fdr = $1.29∙10^{-19}$). The age associated upregulation of kynurenine and downregulation of both serotonin (Cussotto et al., 2020; Miller et al., 2022) and the microbial indole metabolites (Laurans et al., 2018) match the current literature about the role of tryptophan metabolism in inflammaging (inflammation driven aging) and aging.

### 2.6 Acylcarnitine metabolism

The identified acylcarnitines are also known markers of age and aging related diseases (Di Cesare et al., 2022; Jarrell et al., 2020). Decanoyl carnitine was the most important acylcarnitine in our data, and it has also been identified as a female aging predictor in a study by Di Cesare et al. (Di Cesare et al., 2022). Acylcarnitines are positively correlated with a wide range of inflammation driven diseases including type 2 diabetes, cardiovascular diseases, and nonalcoholic fatty liver disease (Dambrova et al., 2022). Finally, acylcarnitines play an essential role in transporting free fatty acids to the mitochondria for the energy metabolism—hence, dysregulation might have a wide array of physiological effects. In coherence with the literature, our identified acylcarnitines increase with age.

### 2.7 Biased compounds

The NN model also included a cocaine derivate, benzoylecgonine, which peaked in concentration at ages 27–29 and had low concentrations for very young and very old samples. Sampling bias caused benzoylecgonine's importance and including it as a predictor allows us to isolate the confounding variables. If omitted, the metabolites affected by cocaine metabolism might become new predictors, and we lose the ability to isolate endogenous aging markers from endogenous cocaine markers. Finally, the exogenous compound, benzoic acid, also appeared as top predictor. Benzoic acid is potentially derived from cocaine, but as it increases throughout life (in contrast to benzoylecgonine) it most likely origins from processed food (Floriani et al., 2014; Leth et al., 2010).

## 3 DISCUSSION

We have used a highly uncontrolled cohort to validate some of the important pathways in aging. Despite the limitations of uncontrolled sample collection, we have shown that routine untargeted measurements are a valuable resource in population‐wide studies of metabolomic biomarkers. The feature selection returned known metabolites that strongly associate with inflammaging, the interplay between inflammation and aging (Franceschi et al., 2018). We also identified a new metabolite, cyclo (leu‐pro) which is not a human metabolite but plays antimicrobial and antiviral roles, and further investigation might elucidate the endogenous effect and the contribution (if any) to accelerated aging or anti‐aging mechanisms.

We identified the tryptophan‐kynurenine metabolism as an aging agent. The literature identifies the enzyme indole‐2,3‐dioxygenase (IDO1) as the center of the tryptophan inflammaging processes (Laurans et al., 2018). IDO1 is upregulated during tryptophan excess (diet/obesity) and in aging individuals and generates kynurenine from tryptophan, while depleting the tryptophan‐based metabolism of serotonin and indoles. Kynurenine binds the Aryl hydrocarbon Receptor (AhR), inducing pro‐inflammatory processes, oxidative processes, and further expression of IDO1 (Kaiser et al., 2020). A moderate AhR activity is beneficial, but high activity is adversarial to health and associates with vascular stiffness, atherosclerosis, and bone mass loss (Esser et al., 2018; B.‐J. Kim et al., 2019; Laurans et al., 2018). Because of these various health impacts, the kynurenine‐tryptophan ratio associates with mortality rates and hazard ratios of elderly individuals in longitudinal studies (Pertovaara et al., 2006; Zuo et al., 2016). Hence, biological age scores might be obtainable by the combination of important pathways instead of using only the RMSE as a proxy of biological age.

The acylcarnitines are also associated with age and aging‐diseases and have been found to increase the hazard ratio of heart attack over a 20 year follow‐up period (Smith et al., 2020). Hence, it makes sense the NN model includes the acylcarnitine pathway and tryptophan derivatives to estimate the biological age. Better data quality may allow biological aging scores based on these two aging pathways exclusively. This would ensure a meaningful age score explaining mortality rather than chronological age as molecules associated with chronological age are not guaranteed to explain mortality (Hertel et al., 2016; L. C. Johnson et al., 2019). Despite this, J. Hertel et al. (2016) showed that the full metabolome correlates with mortality rates—which in turn relates to biological age—using a longitudinal study design (Hertel et al., 2016).

Unfortunately, we were unable to make any direct conclusions about our age‐score (predictions) association to biological age because we lack longitudinal data. Despite this, our error between age‐scores and chronological age (*r* ^*2*^ = 0.63) was on level with the existing literature (Hertel et al., 2016; L. C. Johnson et al., 2019; Menni et al., 2013; Robinson et al., 2020). We also saw that several of the identified compounds were related to inflammatory responses which is likely to reflect biological aging. This shows the potential of using observational metabolomics data in geroscience to obtain generalizing aging scores, independent of, for example, fasting, diet, smoking, and BMI (although we had to remove females because of low sample size).

By combining untargeted data with a large sample size, we obtained the power to find consistent patterns in the untargeted data and to our knowledge only one study has done this before: O. Robinson et. al. used untargeted metabolomics to obtain a state‐of‐the‐art RMSE of 3.7 years (our model: 5.9 years) (Robinson et al., 2020). This performance gap might be caused by experimental variation and sampling bias as our data mainly contain young males unfit for driving. While the experimental variation affects the data quality, the bias impacts our statistics and conclusions. By being aware of the obvious bias, we can exclude biased features from our biological conclusions (e.g., cocaine metabolites). Alternatively, if the biased features are exogenous compounds from, for example, diet or medication, including them as conditionals might help explain their role in aging if they are believed to have any.

The experimental variation is caused by several limitations. First, batch effect is caused by retention time drifts, variation in instrument performance, and change of LC‐columns. Second, noise is caused by time deviances in sample handling from police to TOF‐analysis and by the biologically active whole blood samples that are less appropriate than plasma or serum for this type of analysis. Fourth, protocol updates, including a change of sample tubes in 2020 (see *experimental procedures*), cause differences between years and might explain why the 2020 samples cluster in the PCA (Figure 2b, Figure S1)—we chose not to exclude these samples because we aimed to prove the resource of screening data in metabolomics. Despite the limitations, we consider all samples individually reliable as they were manually approved by a forensics chemist given specifications of internal standards, quality controls, and retention time. It should be noted the data have previously been used for retrospective metabolomics studies about drugs (Nielsen et al., 2016; Wang et al., 2022).

Our use of neural networks entailed several strengths and weaknesses. The model performed better than elastic net and gradient boosting combined with different batch‐normalized datasets. Because we removed the batch normalization step which needs several samples to correct batch effect, our computational setup can predict on a single new sample from the same screening laboratory. Also, our modeling proved to be robust to the strong experimental effects, contributing more than 40% of the variance. Unfortunately, neural networks require large datasets due to the training process (partitioning into test, train, and validation data) and due to the many parameters, making the models susceptible to overfitting.

Neural networks are considered black box models as they are difficult to explain. SHAP values overcome this methodological issue. The industry has already absorbed it to ensure fairness in predictive modeling (e.g., avoiding racial bias), and (high impact) biological studies are beginning to use it as well (Buergel et al., 2022; Weis et al., 2022). We only used the SHAP values to validate whether the neural networks fit followed the linear trends of the raw data (if not, we disregarded the compound), and further investigation might explain the peculiar trends or elucidate the tryptophan and acylcarnitine pathways more profoundly.

We believe that untargeted large‐scale metabolomics provides a strong tool for describing biological age—and that screening data are a valuable data resource—although several aspects can be improved. First, other screening data from the health sector might provide interesting observational data on aging in diseases. Second, modeling time until death might yield more meaningful results for biological aging than modeling chronological age. Third, uncontrolled studies (such as this) contribute with population‐wide aging profiles, making it possible to isolate effects when investigating diseases associated with age.

In summary, this study is a proof of principle under extremely limited experimental conditions, and we have shown that robust methods exploit the large sample size and model age to rescue/extract/identify known biomarkers and potentially new ones. This shows the power of using routine measurements and that large‐scale untargeted studies might become even more informative under better sampling and controlled conditions. Finally, we find that untargeted metabolomics shows a great potential in geroscience, as it monitors a wide range of physiological responses to aging and inflammation.

## 4 EXPERIMENTAL PROCEDURES

### 4.1 Biological material, sample extraction, and untargeted screening

All steps of the data acquisition pipeline have been described in detail in R. Telving et. al. (Telving et al., 2016) and T. Wang et. al (Wang et al., 2022).

In short, whole blood samples were collected by the police given a suspicion of individuals driving under the influence of drugs. The samples were then stored and transported at cooled conditions until receival at the laboratory typically within 2–7 days, and then stored at −18 °C before the analysis (within 1–5 additional days). The analysis was based on ultra‐high‐performance‐liquid‐chromatography quadrupole‐time‐of‐flight mass spectrometry (UHPLC‐QTOF) and was performed on evaporated and reconstituted 30 kDa filtered supernatant from precipitated whole blood as described in detail in Telving et al. (Telving et al., 2016). All samples were manually approved by a forensic chemist given the QCs, internal standards, the retention time were within specifications.

Two large changes in the laboratory protocol were implemented during the sampling period from 2017 to 2020. First, the sample tubes were changed from FC (sodium fluoride, sodium EDTA, citric acid, sodium citrate; pH 5.9) to FX (sodium fluoride, potassium oxalate; pH 7.4). Second, the sample extraction procedure was changed from a manual to a semi‐automated procedure (30‐10‐2018) which is most likely not affecting the data quality negatively.

For identification purposes, fragmentation analysis was carried out using broadband Collision Induced Dissociation (bbCID) with a collision energy of 25 eV. Auto‐MS/MS with collisions at energies from 10 to 35 eV was additionally used for verification of cyclo (leu‐pro) in samples compared to a 0.1 μg/mL standard solution cyclo (leu‐pro), abcr GmbH, Germany. All other discussed compounds had previously been identified and existed in our inhouse library.

### 4.2 Preprocessing using XCMS

11,171 mzML files were preprocessed using an XCMS (ver. 3.8.0) workflow tailored for parallel processing on a high‐performance computing cluster. The 11,171 files included both male and female samples. Peak picking and peak integration (including imputation of missing values) were performed on single files in parallel, resulting in a total run time of 4–5 days and peak memory consumption of 102 GB, which is less than the size of the full data set (>5 TB). The grouping and alignment happened simultaneously on all files in standard XCMS functions. Table S1 describes the steps of the workflow—peak identification, alignment, grouping, and filling—and the XCMS parameter settings.

### 4.3 Preprocessing methods

Before experimental effect correction, the feature intensities were fourth root transformed and any female samples were removed. Samples that were extreme outliers were removed if having PCA scores more than 1.5*95th quantile away from the median PCA score in any of the first 12 components. Further, features were removed if missing in more than 20% of the samples in any batch. The sample outlier removal step reduced the data from 10,133 to 9929 samples, and the feature removal reduced the count from 12,686 to 8038 features.

The chosen normalization methods were WaveICA2.0, Combat, quantile normalization (Limma ver. 3.42.0), and robust row normalization.

The robust row normalization was performed by dividing sample values by the sample sums of a subset of robust features. The subset (“robust features”) was selected by two criteria: First, if the median feature rank across all samples was between the 20th and 80th quantile of all features' median ranks; and second, if the difference of min and max rank of a feature was between the 20th and 80th quantile of all min‐max differences. This would ensure robust features that do not fluctuate much from run to run. The number of robust features amounted to 4115.

### 4.4 Modeling

The data used for modeling were randomly partitioned into train (80%, *n* = 7943), development (10%, *n* = 993), and test (10%, *n* = 993). The development partition was only used to evaluate the NN model during training. Hence, the model screening of the simple models (see below) used both the train and development partition to fit the models. The test partition was used *only* for the final validation of the models.

#### 4.4.1 Model screening

A model screening of elastic net (glmnet ver. 4.0.2) and gradient boosting (xgbTree ver. 1.2.0) was performed on the normalized data sets to find the best combination of normalization and machine learning model (Chen & Guestrin, 2016; Zou & Hastie, 2005). The models were tuned and evaluated using a fivefold cross validation in the R package, caret (ver. 6.0.86), with the *tuneLength* set to 5 for hyperparameter tuning (Kuhn, 2008). For the model screening, only the training and development partitions were used, and the models were selected to cover linear and tree‐based models with varying tendency to overfit. The best preprocessing‐model combination was evaluated on the test set to achieve an unbiased performance estimate.

#### 4.4.2 Neural networks model using ratios

To improve the statistical analysis and following interpretation, the data must be normalized to correct for experimental noise. Existing normalization methods neglect the normalizing potential of compound ratios, so we implemented a ratio‐based NN model. This NN model was compared against quantile normalization, Combat (W. E. Johnson et al., 2007), WaveICA2.0 (Deng et al., 2021), row normalization (see *experimental procedures*), and fourth root transformed raw data.

Studies have shown that ratios work well for normalizing the biomarkers because ratios follow the same distribution across samples, as opposed to single compound intensities which depend on, for example, sample quality and batch effect (Petersen et al., 2012). With untargeted screening data, thousands of features yield millions of ratios making brute force approaches extremely inefficient computationally and sensitive to spurious correlations (i.e., ratios that randomly correlate with a feature of interest but are impossible to validate). Consequently, we present a ratio‐based neural network that implements the anticipation of the self‐normalizing effect of ratios, by selecting only a small fraction of informative features used for the ratios (Figure 1).

A neural network consists of layers that each contain multiple linear models. The linear models of the first layer use the feature intensities of a sample as input. During training, each linear model is fitted to find a pattern in the feature intensities that improves the final prediction. For instance, one linear model might use features that correlate with age and another linear model might use features that explain the baseline intensity of the sample. The following layer in the neural network also consists of linear models, and these models use the outputs from the first layer to combine the information, that is, age‐correlated features and features explaining baseline intensity (Figure S8 and Figure S9).

By introducing ratios between the first layer and the second layer, we can force the output of one linear model (e.g., age score) to be divided by the output of another linear model (e.g., a baseline score). We can imagine that a high‐quality sample will have its age score divided by a high baseline score, and vice versa for low quality samples. This will normalize the age scores between samples with different baselines. In principle, the model outputs of the first layer could also represent two different pathways, single compounds, noise levels, etc., conditioned that the output benefits the final prediction accuracy. In practice, it is most likely the penalized linear models find the age‐correlated features and some stable features that reflect the state of the sample.

#### 4.4.3 NN Architecture

Pytorch (ver. 1.10.1) was used for the implementation (Paszke et al., 2017). The architecture and hyperparameters of the NN model were decided by a grid search. The best model consisted of one input layer and one ratio layer followed by a dense neural network of two hidden layers and an output layer (Table 2). The Adam optimizer was used with a learning rate of 3e‐4 on batches of 500 samples and a total of 1000 epochs to ensure convergence (due to unstable learning). All layers, except the ratio layer, used a dropout of 0.1. The model can be found at https://github.com/JohanLassen/untargeted_ratios—it is meant as a building block for NN implementations using ratios, not as a plug‐n‐play implementation.

**TABLE 2:** Neural network model specifications.

Layer	Specifications	Nodes
Linear layer	Activation function: None Batch normalization^a^: None weight decay: 0.5	12
Ratio layer	Unique ratios of layer 1 outputs. Activation function: ReLU Batch normalization^a^: Yes weight decay: None (no weights)	66 ratios (12^a^ (12–1))/2 = 66
Linear layer	Activation function: ReLU Batch normalization^a^: Yes weight decay: 0.1	33
Linear layer	Activation function: ReLU Batch normalization^a^: Yes weight decay: 0.01	33
Output layer	1 node weight decay: *default* Outputs prediction	1

a Batch normalization in neural networks refers to the normalization of model weights during training and not normalization of the feature data.

The NN model was fitted on the training data (80% of the data) and evaluated during the training on the development data. A total of 1000 NN models (using the final architecture, Table 2) were fitted, and their performance was evaluated on the held‐out test data. The reported model performance was the RMSE of the mean values of each predicted observation, which ensured good repeatability of the neural network performance.

#### 4.4.4 Feature Importance

SHAP values were calculated from the 1000 fitted pyTorch models using the SHAP package (Lundberg & Lee, 2017). This generated 1000 Shapley value matrices (of n observations and p features) which were averaged to one matrix of the same size (n observations and p features). Following this, global importance was calculated by taking the variance of each feature. SHAP values are upcoming in the metabolomics field and applicable in a large range of different models (Buergel et al., 2022; Weis et al., 2022).

### 4.5 Feature annotation

The features were annotated by metID (Shen et al., 2022) with use of the databases: massbank, mona, NIST, msdatabase, orbitrap, hmdb, the fiehn hilic database, and our own in‐house database of approximately 400 metabolites. All features discussed in the manuscript have been manually quality assured, while non‐significant hits have not been fully assessed (included in supplementary, all reference spectra in Data S2). All discussed compounds are ID level 1 matched by m/z, fragmentation pattern, and retention time against our inhouse library (following the guidelines of Metabolomics Standard Initiative) (Sumner et al., 2007) (annotations in Data S1, reference spectra in Data S2). Among the top 5 features, we excluded one annotation, M255T346, which matched poorly to the 6‐dehydrotestosterone glucuronide reference spectra (data not shown).

## AUTHOR CONTRIBUTIONS

The study was conceived by JKL and PV. Samples were provided by MJ and JBH. Data analysis was performed by JKL, TW, and PV. The manuscript was drafted by JKL and PV and reviewed by JBH, MJ, TW, and KLN. The final version was approved by all authors.

## CONFLICT OF INTEREST STATEMENT

The authors declare they have no conflicts of interest.

## Supporting information


Data S1:

Data S2:

Appendix S1.

## Data Availability

Data sharing must comply with GDPR meaning published data must be anonymized, and the pseudo anonymized data (used for the analysis) are deleted once the study reaches its end.
