# Supplementary material for: Large‐Scale metabolomics: Predicting biological age using 10,133 routine untargeted LC–MS measurements
Source: Aging Cell. 2023 Mar 19;22(5):e13813. doi: 10.1111/acel.13813 (PMC10186604; doi:10.1111/acel.13813)

Cyclo(leu-pro) identification

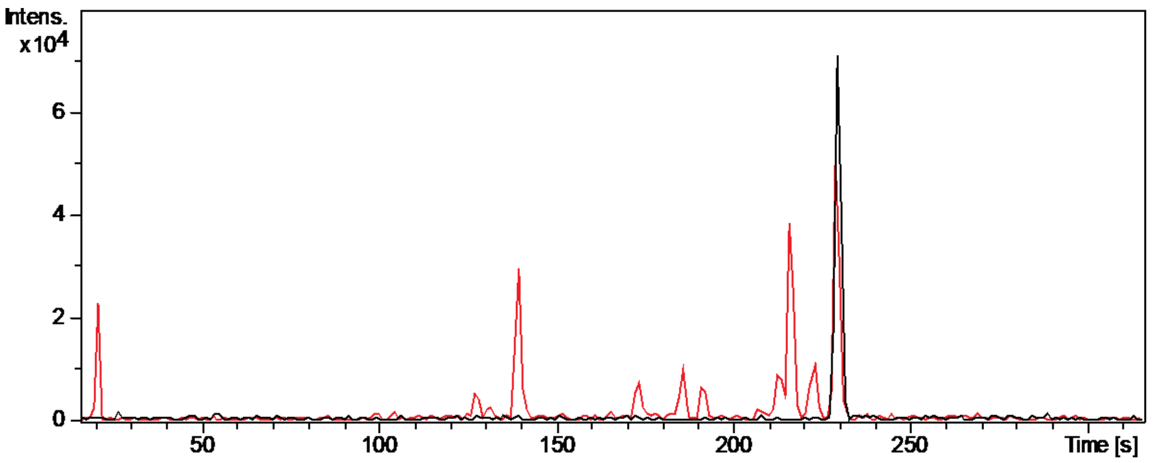

EIC of:
Black = standard compound cyclo(leu-pro)
Red = blood sample

RT = 229 sec

MS/MS Spectrum of standard compound

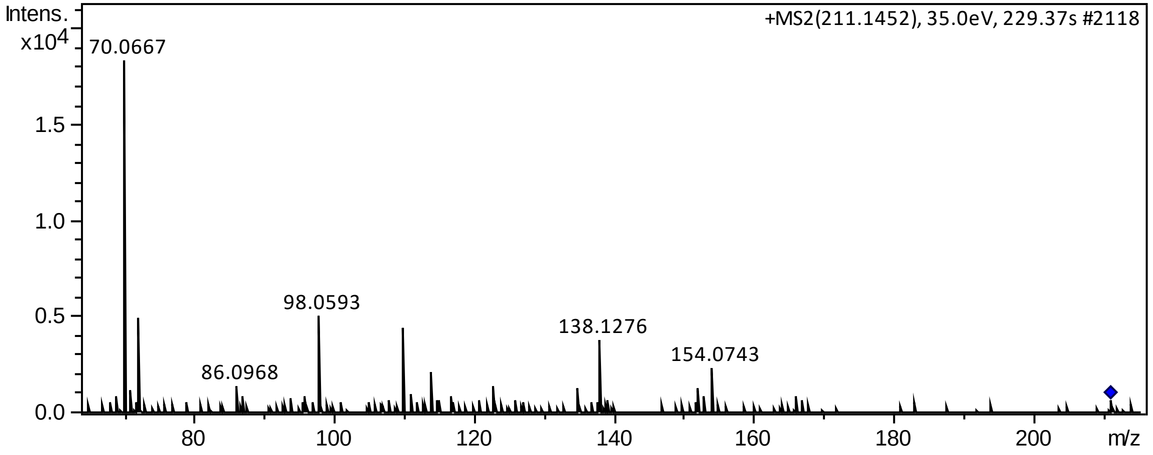

MS/MS Spectrum of 211.1442 in blood sample

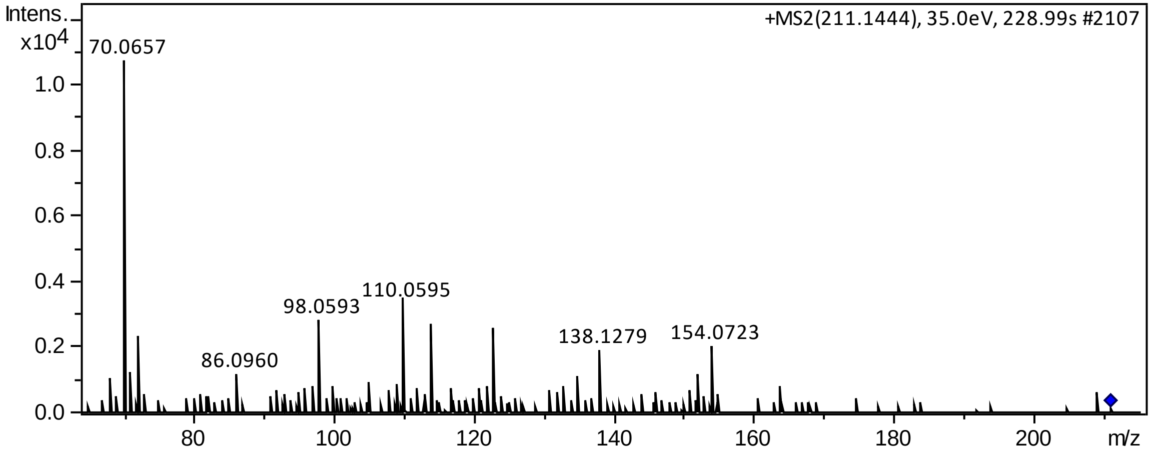

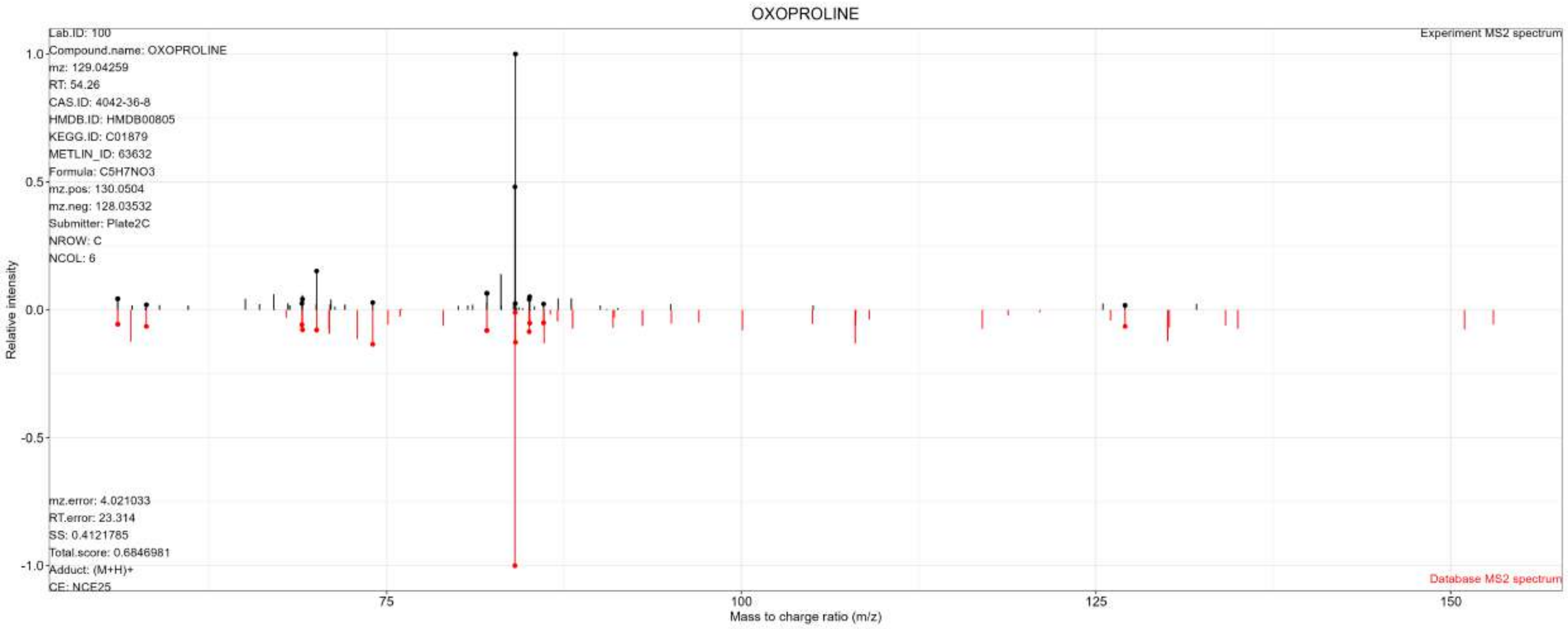
OXOPROLINE
Lab.ID: 100
Compound.name: OXOPROLINE
mz: 129.04259
RT: 54.26
CAS.ID: 4042-36-8
HMDB.ID: HMDB00805
KEGG.ID: C01879
METLIN_ID: 63632
Formula: C5H7NO3
mz.pos: 130.0504
mz.neg: 128.03532
Submitter: Plate2C
NROW: C
NCOL: 6
mz.error: 4.021033
RT.error: 23.314
SS: 0.4121785
Total.score: 0.6846981
Adduct: (M+H)+
CE: NCE25
Experiment MS2 spectrum
Database MS2 spectrum
Relative intensity
1.0
0.5
0.0
-0.5
-1.0
Mass to charge ratio (m/z)
75
100
125
150

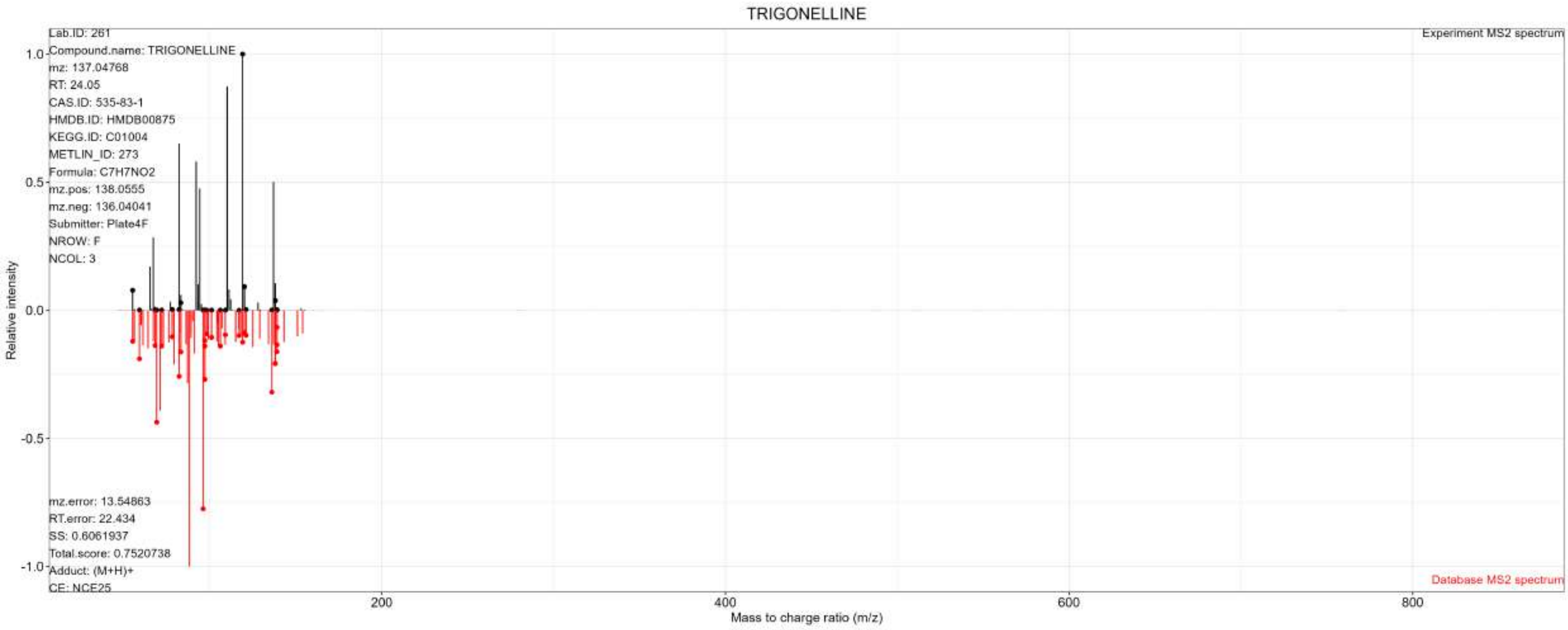
TRIGONELLINE
Lab.ID: 261
Compound.name: TRIGONELLINE
mz: 137.04768
RT: 24.05
CAS.ID: 535-83-1
HMDB.ID: HMDB00875
KEGG.ID: C01004
METLIN_ID: 273
Formula: C7H7NO2
mz.pos: 138.0555
mz.neg: 136.04041
Submitter: Plate4F
NROW: F
NCOL: 3
mz.error: 13.54863
RT.error: 22.434
SS: 0.6061937
Total.score: 0.7520738
Adduct: (M+H)+
CE: NCE25
Experiment MS2 spectrum
Database MS2 spectrum
Relative intensity
1.0
0.5
0.0
-0.5
-1.0
200
400
600
800
Mass to charge ratio (m/z)

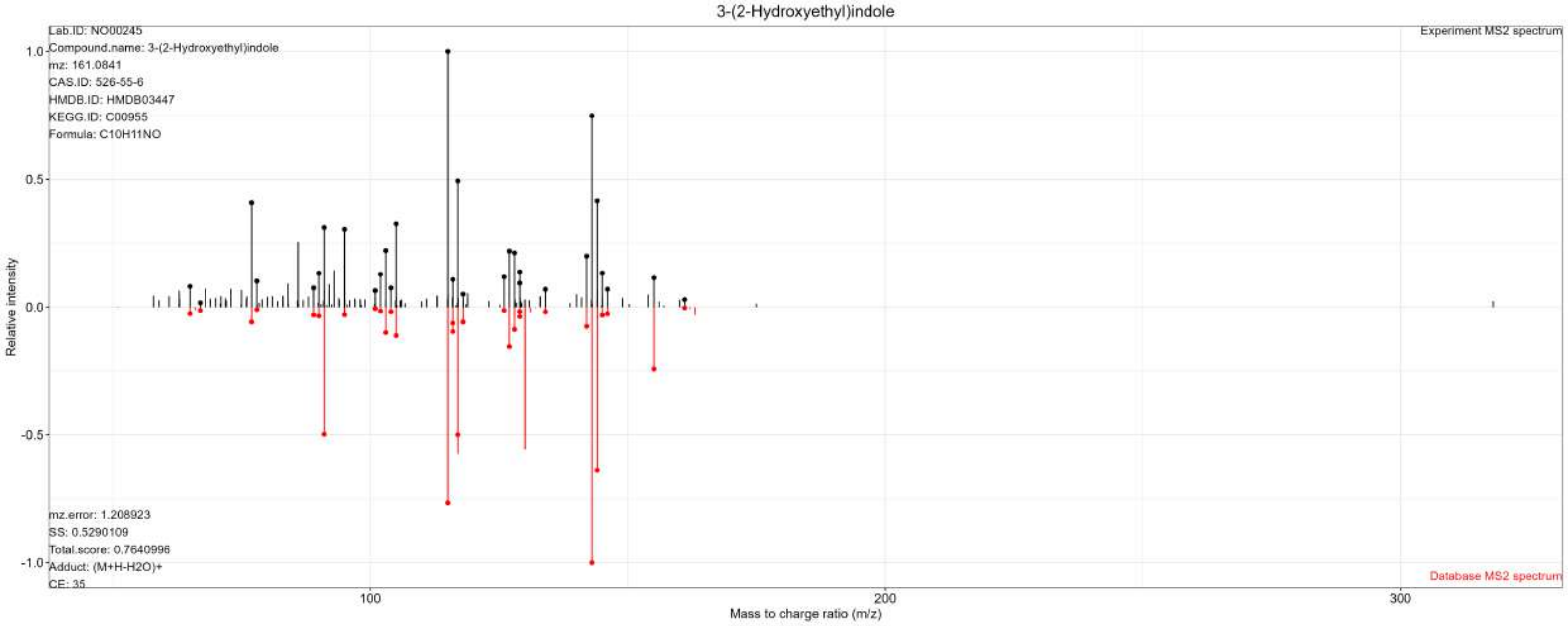
3-(2-Hydroxyethyl)indole
Lab.ID: NO00245
Compound.name: 3-(2-Hydroxyethyl)indole
mz: 161.0841
CAS.ID: 526-55-6
HMDB.ID: HMDB03447
KEGG.ID: C00955
Formula: C10H11NO
Experiment MS2 spectrum
mz.error: 1.208923
SS: 0.5290109
Total.score: 0.7640996
Adduct: (M+H-H2O)+
CE: 35
Database MS2 spectrum
Relative intensity
Mass to charge ratio (m/z)
1.0
0.5
0.0
-0.5
-1.0
100
200
300

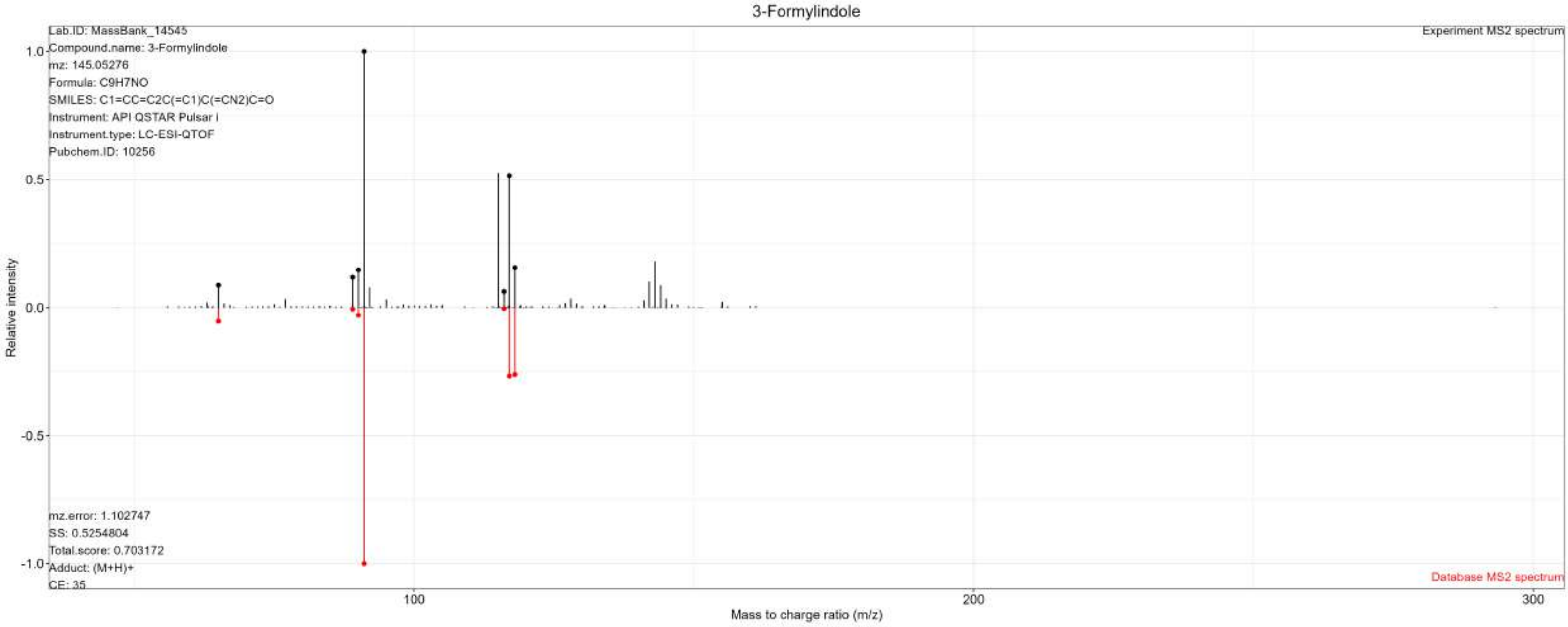
3-Formylindole
Lab.ID: MassBank_14545
Compound.name: 3-Formylindole
mz: 145.05276
Formula: C9H7NO
SMILES: C1=CC=C2C(=C1)C(=CN2)C=O
Instrument: API QSTAR Pulsar i
Instrument.type: LC-ESI-QTOF
Pubchem.ID: 10256
Experiment MS2 spectrum
mz.error: 1.102747
SS: 0.5254804
Total.score: 0.703172
Adduct: (M+H)+
CE: 35
Database MS2 spectrum
Relative intensity
Mass to charge ratio (m/z)
1.0
0.5
0.0
-0.5
-1.0
100
200
300

INDOLE-3-ACETATE

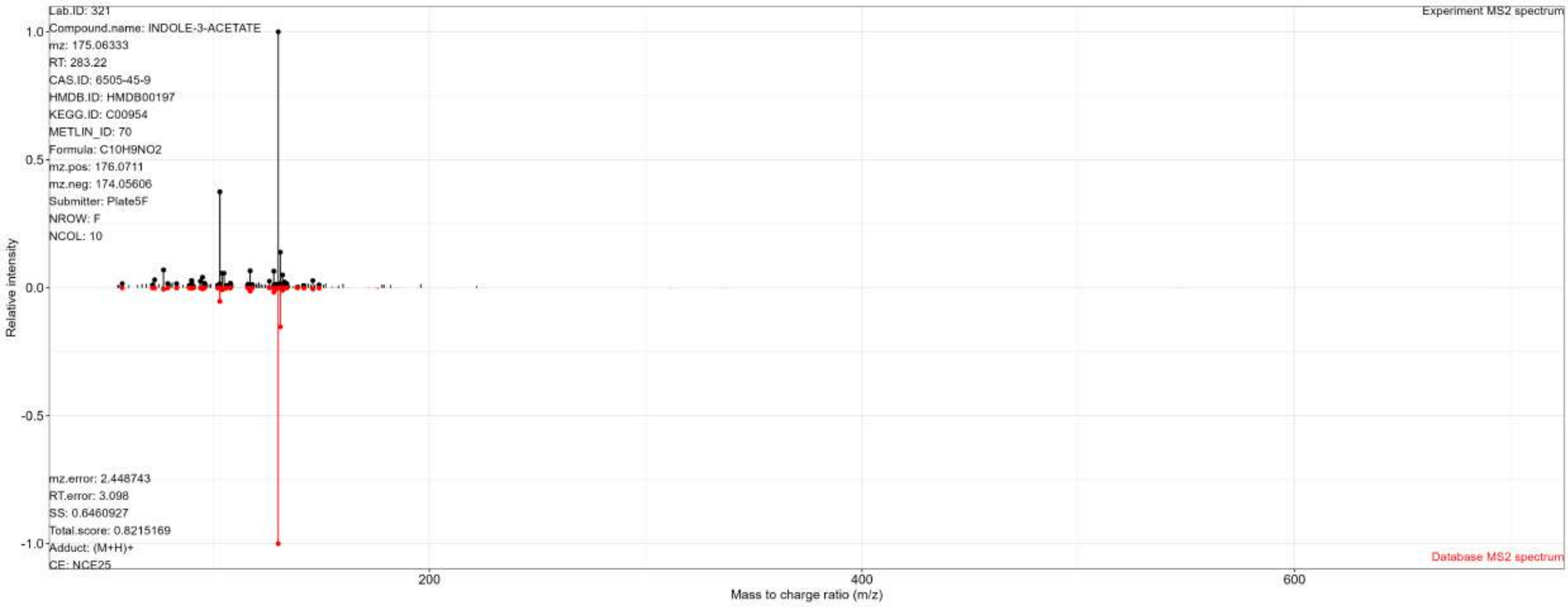

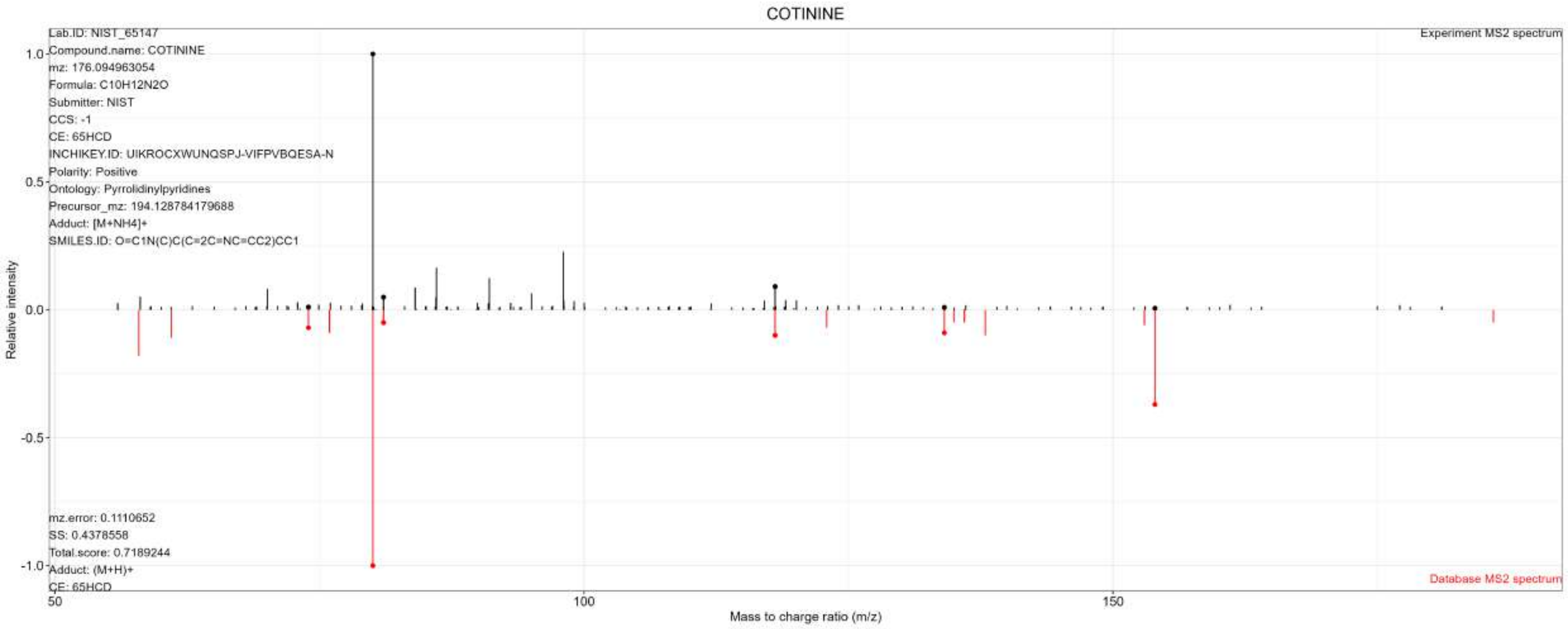
COTININE
Lab.ID: NIST_65147
Compound.name: COTININE
mz: 176.094963054
Formula: C10H12N2O
Submitter: NIST
CCS: -1
CE: 65HCD
INCHIKEY.ID: UIKROCXWUNQSPJ-VIFPVBQESA-N
Polarity: Positive
Ontology: Pyrrolidinylpyridines
Precursor_mz: 194.128784179688
Adduct: [M+NH4]+
SMILES.ID: O=C1N(C)C(C=2C=NC=CC2)CC1
Experiment MS2 spectrum
mz.error: 0.1110652
SS: 0.4378558
Total.score: 0.7189244
Adduct: (M+H)+
CE: 65HCD
Database MS2 spectrum
Relative intensity
1.0
0.5
0.0
-0.5
-1.0
50
100
150
Mass to charge ratio (m/z)

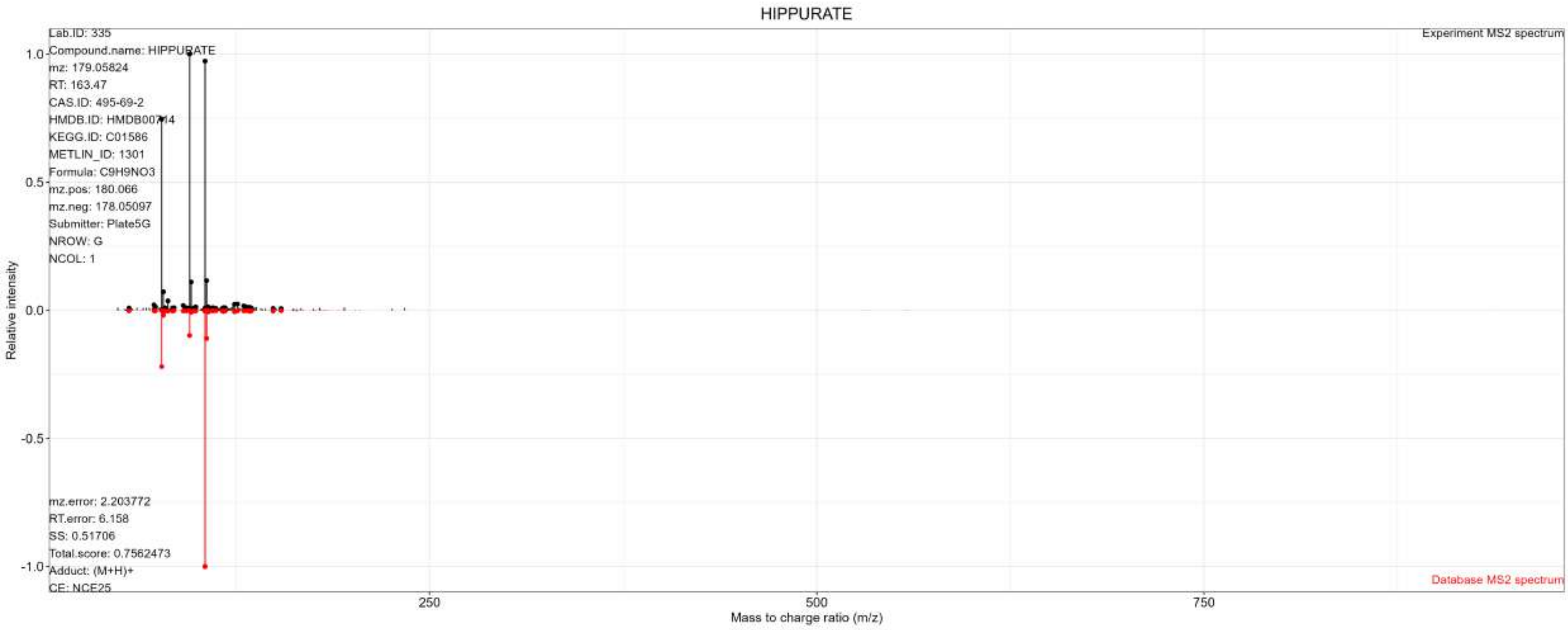
HIPPURATE
Lab.ID: 335
Compound.name: HIPPURATE
mz: 179.05824
RT: 163.47
CAS.ID: 495-69-2
HMDB.ID: HMDB00714
KEGG.ID: C01586
METLIN_ID: 1301
Formula: C9H9NO3
mz.pos: 180.066
mz.neg: 178.05097
Submitter: Plate5G
NROW: G
NCOL: 1
mz.error: 2.203772
RT.error: 6.158
SS: 0.51706
Total.score: 0.7562473
Adduct: (M+H)+
CE: NCE25
Experiment MS2 spectrum
Database MS2 spectrum
Relative intensity
Mass to charge ratio (m/z)
1.0
0.5
0.0
-0.5
-1.0
250
500
750

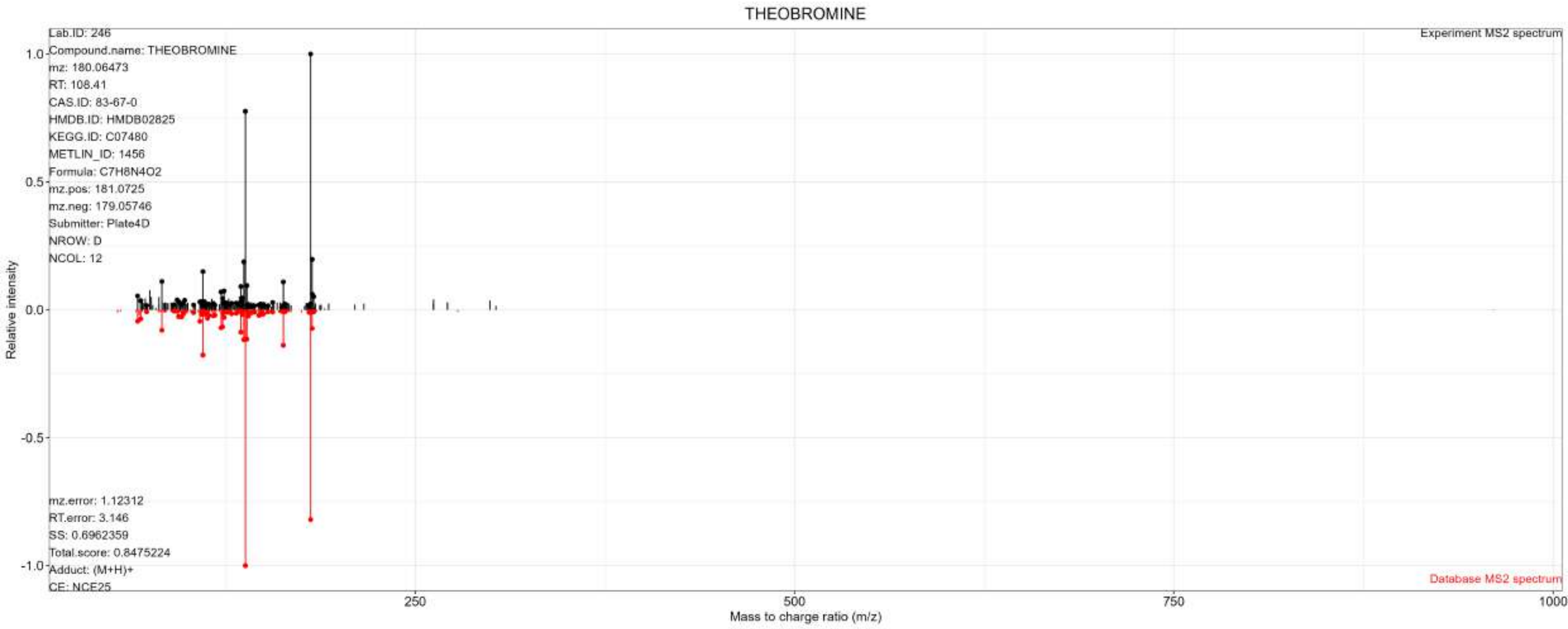
THEOBROMINE
Lab.ID: 246
Compound.name: THEOBROMINE
mz: 180.06473
RT: 108.41
CAS.ID: 83-67-0
HMDB.ID: HMDB02825
KEGG.ID: C07480
METLIN_ID: 1456
Formula: C7H8N4O2
mz.pos: 181.0725
mz.neg: 179.05746
Submitter: Plate4D
NROW: D
NCOL: 12
mz.error: 1.12312
RT.error: 3.146
SS: 0.6962359
Total.score: 0.8475224
Adduct: (M+H)+
CE: NCE25
Experiment MS2 spectrum
Database MS2 spectrum
Relative intensity
Mass to charge ratio (m/z)
1.0
0.5
0.0
-0.5
-1.0
250
500
750
1000

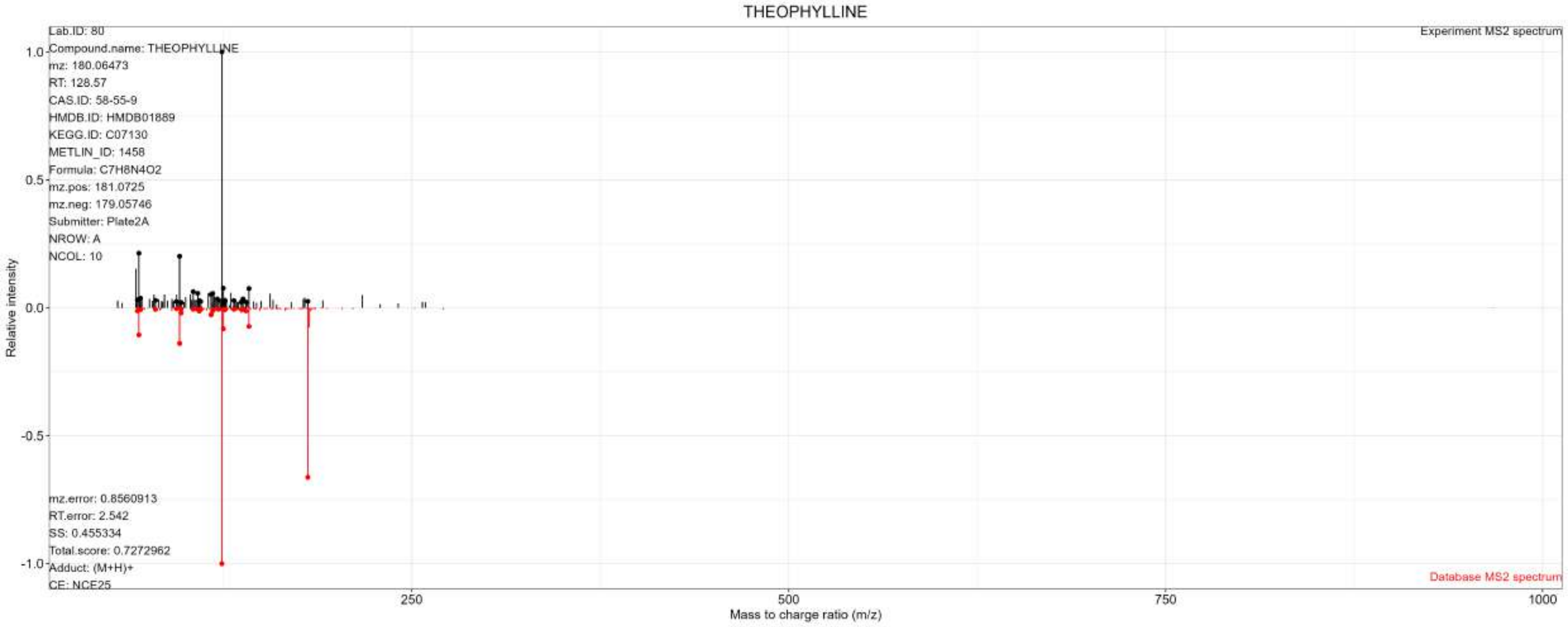

THEOPHYLLINE
Lab.ID: 80
Compound.name: THEOPHYLLINE
mz: 180.06473
RT: 128.57
CAS.ID: 58-55-9
HMDB.ID: HMDB01889
KEGG.ID: C07130
METLIN_ID: 1458
Formula: C7H8N4O2
mz.pos: 181.0725
mz.neg: 179.05746
Submitter: Plate2A
NROW: A
NCOL: 10
mz.error: 0.8560913
RT.error: 2.542
SS: 0.455334
Total.score: 0.7272962
Adduct: (M+H)+
CE: NCE25
Experiment MS2 spectrum
Database MS2 spectrum
Relative intensity
Mass to charge ratio (m/z)
1.0
0.5
0.0
-0.5
-1.0
250
500
750
1000

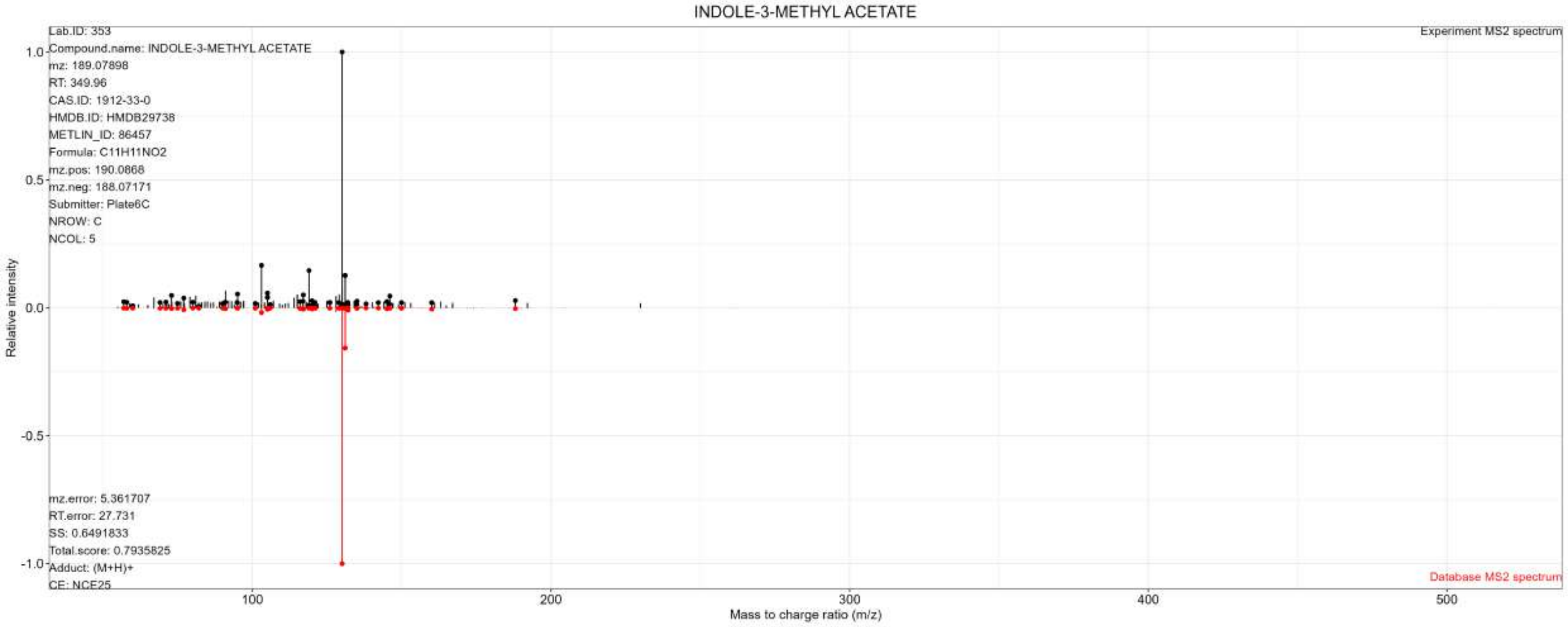
INDOLE-3-METHYL ACETATE
Lab.ID: 353
Compound.name: INDOLE-3-METHYL ACETATE
mz: 189.07898
RT: 349.96
CAS.ID: 1912-33-0
HMDB.ID: HMDB29738
METLIN_ID: 86457
Formula: C11H11NO2
mz.pos: 190.0868
mz.neg: 188.07171
Submitter: Plate6C
NROW: C
NCOL: 5
mz.error: 5.361707
RT.error: 27.731
SS: 0.6491833
Total.score: 0.7935825
Adduct: (M+H)+
CE: NCE25
Experiment MS2 spectrum
Database MS2 spectrum
Relative intensity
Mass to charge ratio (m/z)
1.0
0.5
0.0
-0.5
-1.0
100
200
300
400
500

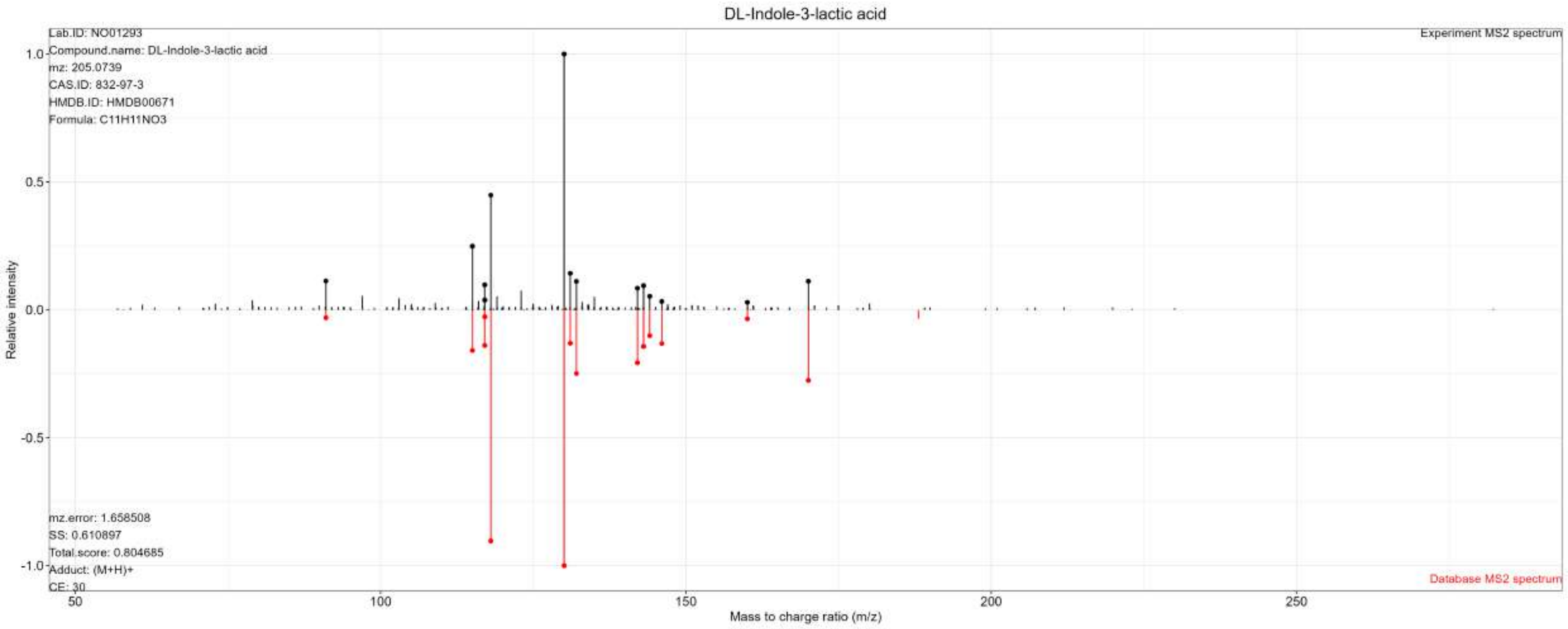

DL-Indole-3-lactic acid
Lab.ID: NO01293
Compound.name: DL-Indole-3-lactic acid
mz: 205.0739
CAS.ID: 832-97-3
HMDB.ID: HMDB00671
Formula: C11H11NO3
Experiment MS2 spectrum
mz.error: 1.658508
SS: 0.610897
Total.score: 0.804685
Adduct: (M+H)+
CE: 30
Database MS2 spectrum
Relative intensity
Mass to charge ratio (m/z)

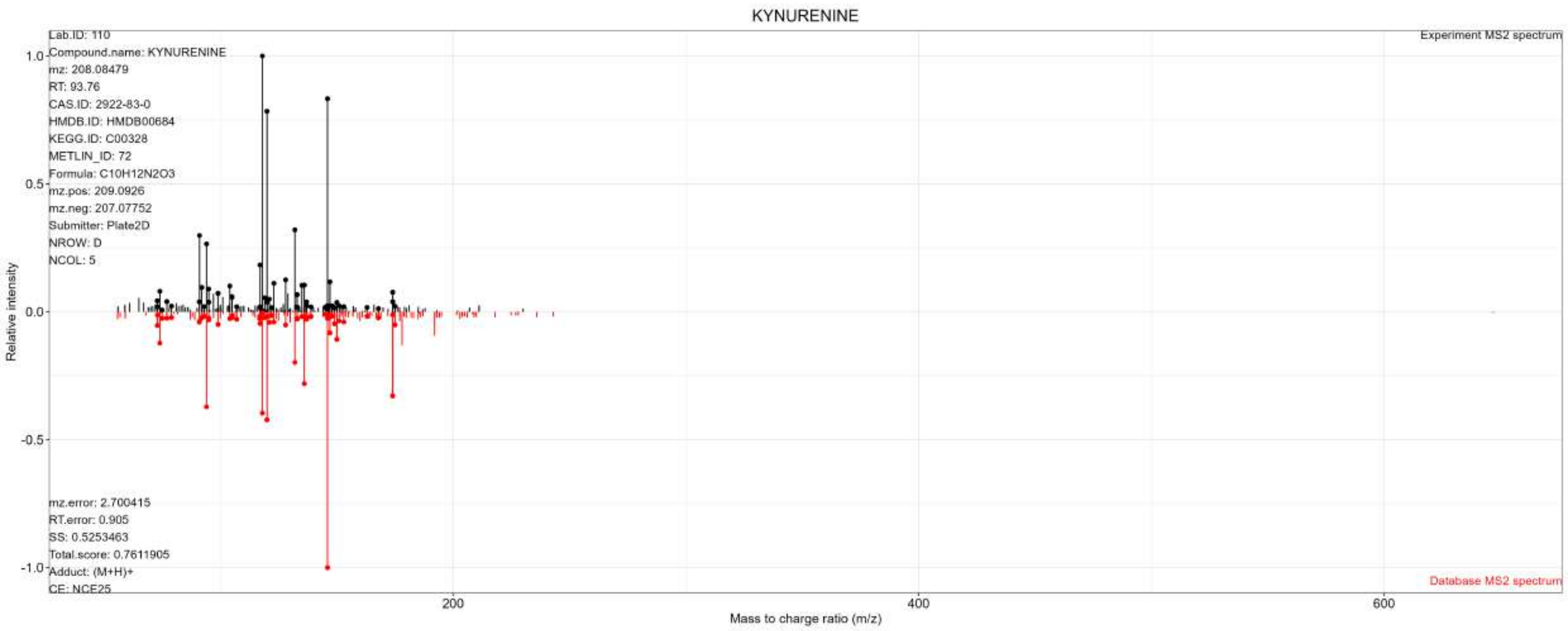
KYNURENINE
Lab.ID: 110
Compound.name: KYNURENINE
mz: 208.08479
RT: 93.76
CAS.ID: 2922-83-0
HMDB.ID: HMDB00684
KEGG.ID: C00328
METLIN_ID: 72
Formula: C10H12N2O3
mz.pos: 209.0926
mz.neg: 207.07752
Submitter: Plate2D
NROW: D
NCOL: 5
mz.error: 2.700415
RT.error: 0.905
SS: 0.5253463
Total.score: 0.7611905
Adduct: (M+H)+
CE: NCE25
Experiment MS2 spectrum
Database MS2 spectrum
Relative intensity
Mass to charge ratio (m/z)
1.0
0.5
0.0
-0.5
-1.0
200
400
600

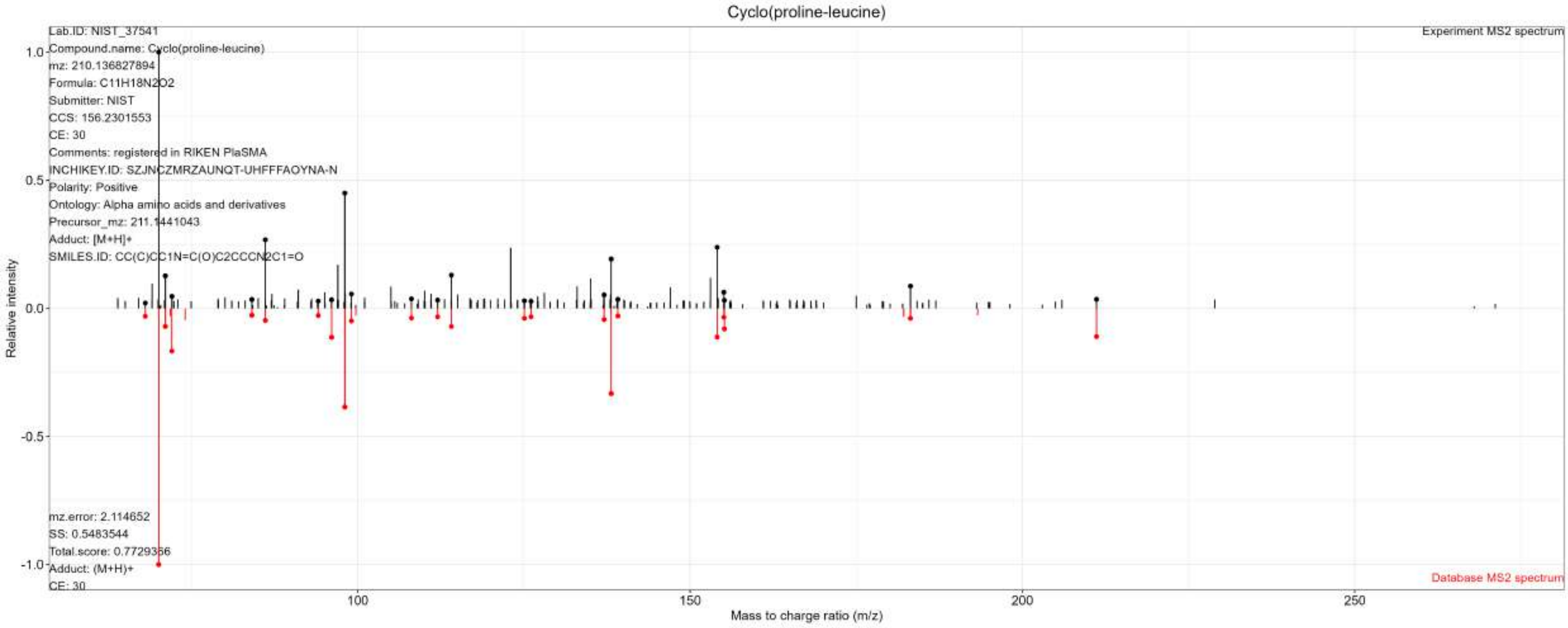

Cyclo(proline-leucine)
Lab.ID: NIST_37541
Compound.name: Cyclo(proline-leucine)
mz: 210.136827894
Formula: C11H18N2O2
Submitter: NIST
CCS: 156.2301553
CE: 30
Comments: registered in RIKEN PlaSMA
INCHIKEY.ID: SZJNCZMRZAUNQT-UHFFFAOYNA-N
Polarity: Positive
Ontology: Alpha amino acids and derivatives
Precursor_mz: 211.1441043
Adduct: [M+H]+
SMILES.ID: CC(C)CC1N=C(O)C2CCCN2C1=O
Experiment MS2 spectrum
mz.error: 2.114652
SS: 0.5483544
Total.score: 0.7729356
Adduct: (M+H)+
CE: 30
Database MS2 spectrum
Relative intensity
Mass to charge ratio (m/z)

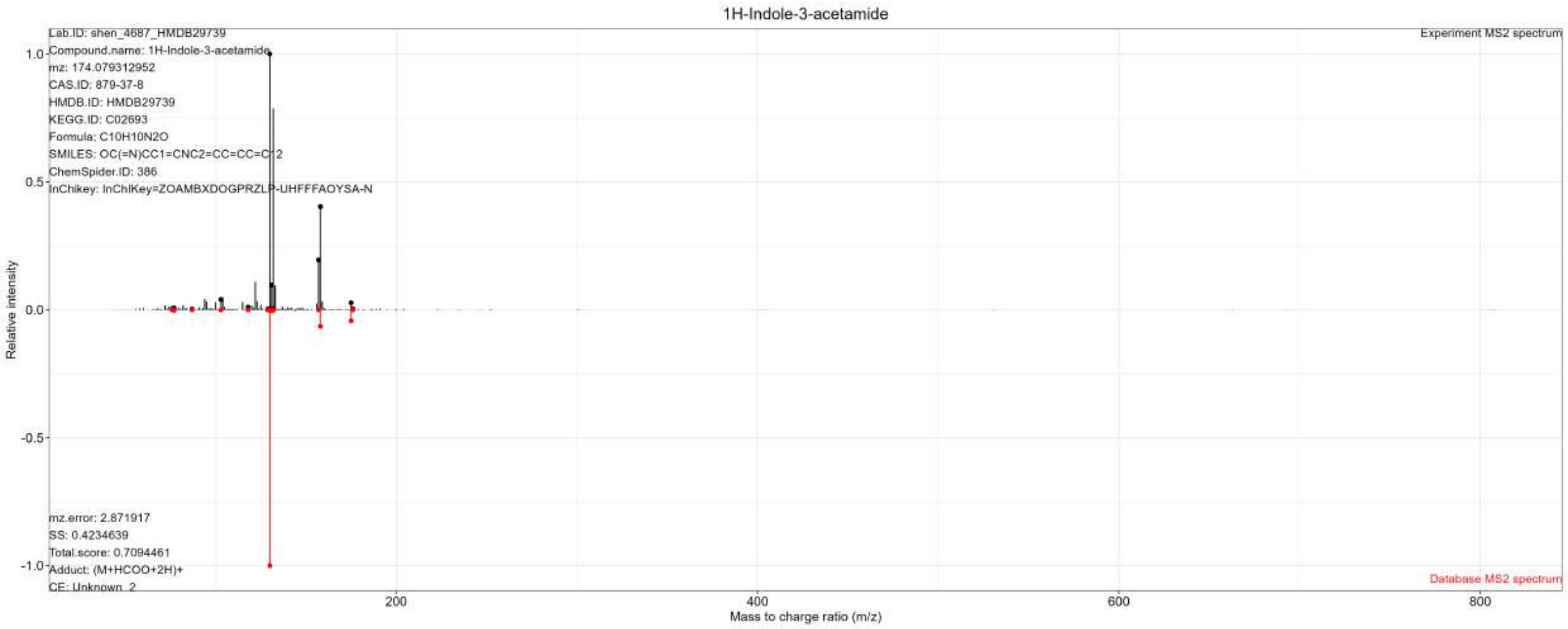
1H-Indole-3-acetamide
Lab.ID: shen_4687_HMDB29739
Compound.name: 1H-Indole-3-acetamide
mz: 174.079312952
CAS.ID: 879-37-8
HMDB.ID: HMDB29739
KEGG.ID: C02693
Formula: C10H10N2O
SMILES: OC(=N)CC1=CNC2=CC=CC=C12
ChemSpider.ID: 386
InChikey: InChIKey=ZOAMBXDOGPRZLP-UHFFFAOYSA-N
Experiment MS2 spectrum
mz.error: 2.871917
SS: 0.4234639
Total.score: 0.7094461
Adduct: (M+HCOO+2H)+
CE: Unknown_2
Database MS2 spectrum
Relative intensity
Mass to charge ratio (m/z)
1.0
0.5
0.0
-0.5
-1.0
200
400
600
800

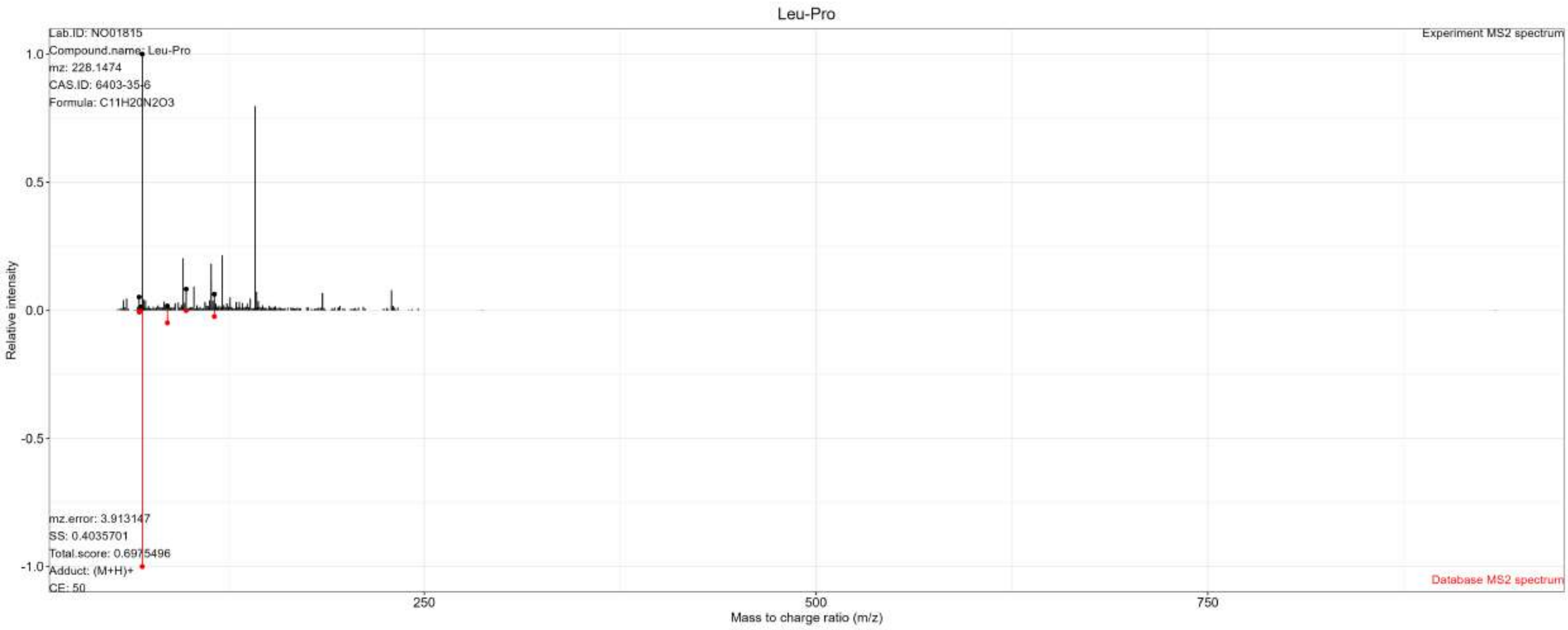

Leu-Pro
Lab.ID: NO01815
Compound.name: Leu-Pro
mz: 228.1474
CAS.ID: 6403-35-6
Formula: C11H20N2O3
Experiment MS2 spectrum
mz.error: 3.913147
SS: 0.4035701
Total.score: 0.6975496
Adduct: (M+H)+
CE: 50
Database MS2 spectrum
Relative intensity
Mass to charge ratio (m/z)

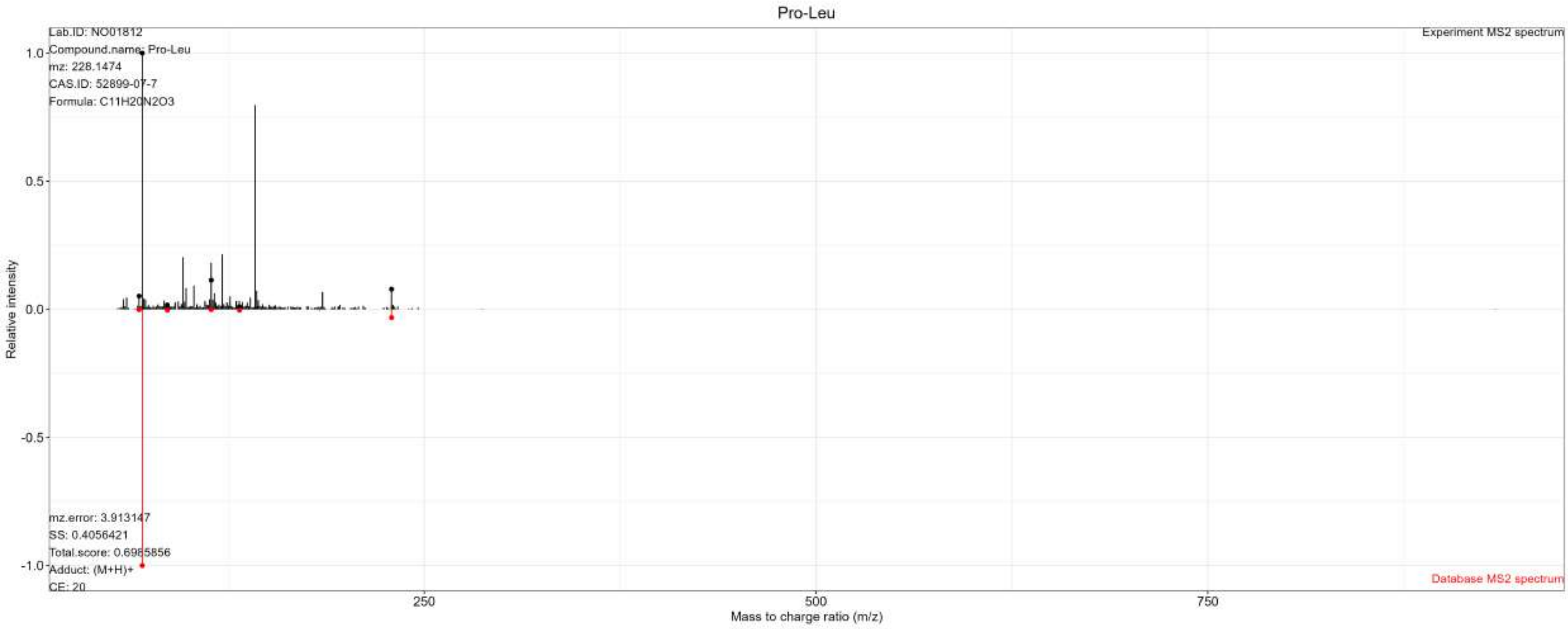

Pro-Leu
Lab.ID: NO01812
Compound.name: Pro-Leu
mz: 228.1474
CAS.ID: 52899-07-7
Formula: C11H20N2O3
Experiment MS2 spectrum
mz.error: 3.913147
SS: 0.4056421
Total.score: 0.6985856
Adduct: (M+H)+
CE: 20
Database MS2 spectrum
Relative intensity
Mass to charge ratio (m/z)
1.0
0.5
0.0
-0.5
-1.0
250
500
750

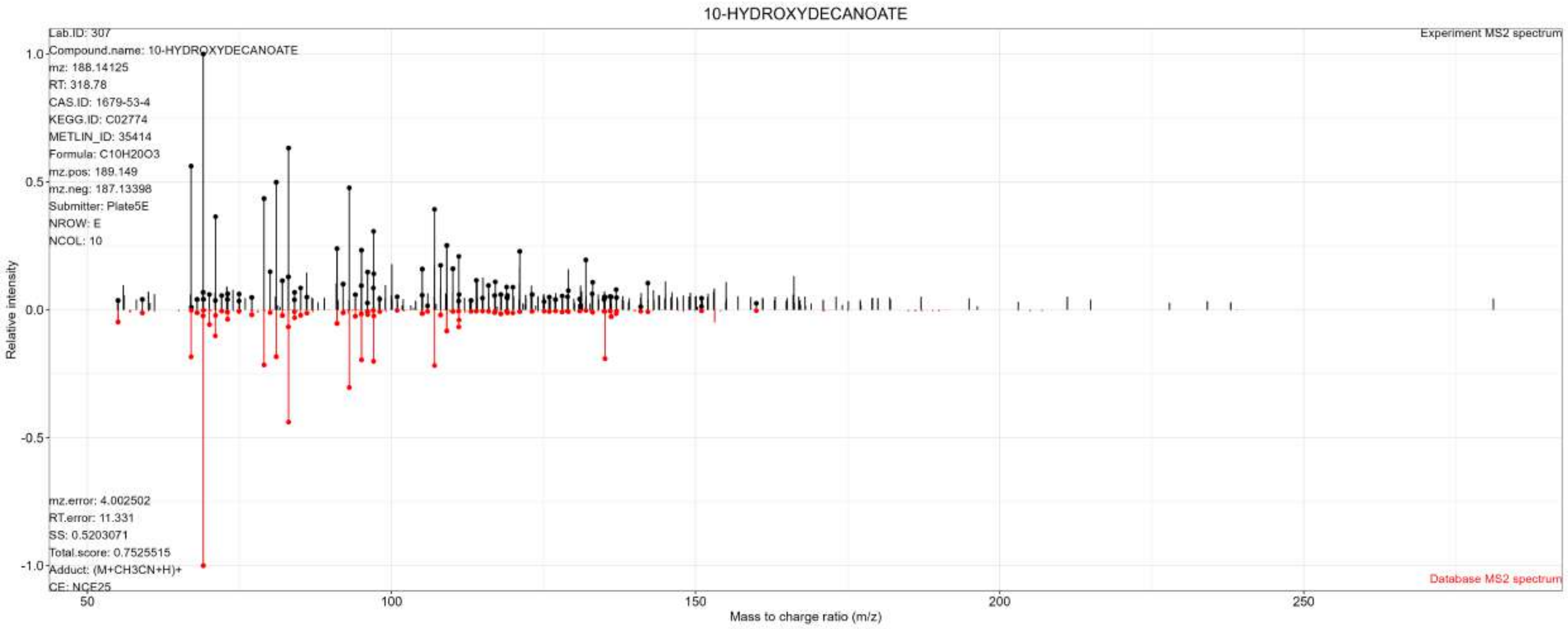
10-HYDROXYDECANOATE
Lab.ID: 307
Compound.name: 10-HYDROXYDECANOATE
mz: 188.14125
RT: 318.78
CAS.ID: 1679-53-4
KEGG.ID: C02774
METLIN_ID: 35414
Formula: C10H20O3
mz.pos: 189.149
mz.neg: 187.13398
Submitter: Plate5E
NROW: E
NCOL: 10
mz.error: 4.002502
RT.error: 11.331
SS: 0.5203071
Total.score: 0.7525515
Adduct: (M+CH3CN+H)+
CE: NCE25
Experiment MS2 spectrum
Database MS2 spectrum
Relative intensity
Mass to charge ratio (m/z)
1.0
0.5
0.0
-0.5
-1.0
50
100
150
200
250

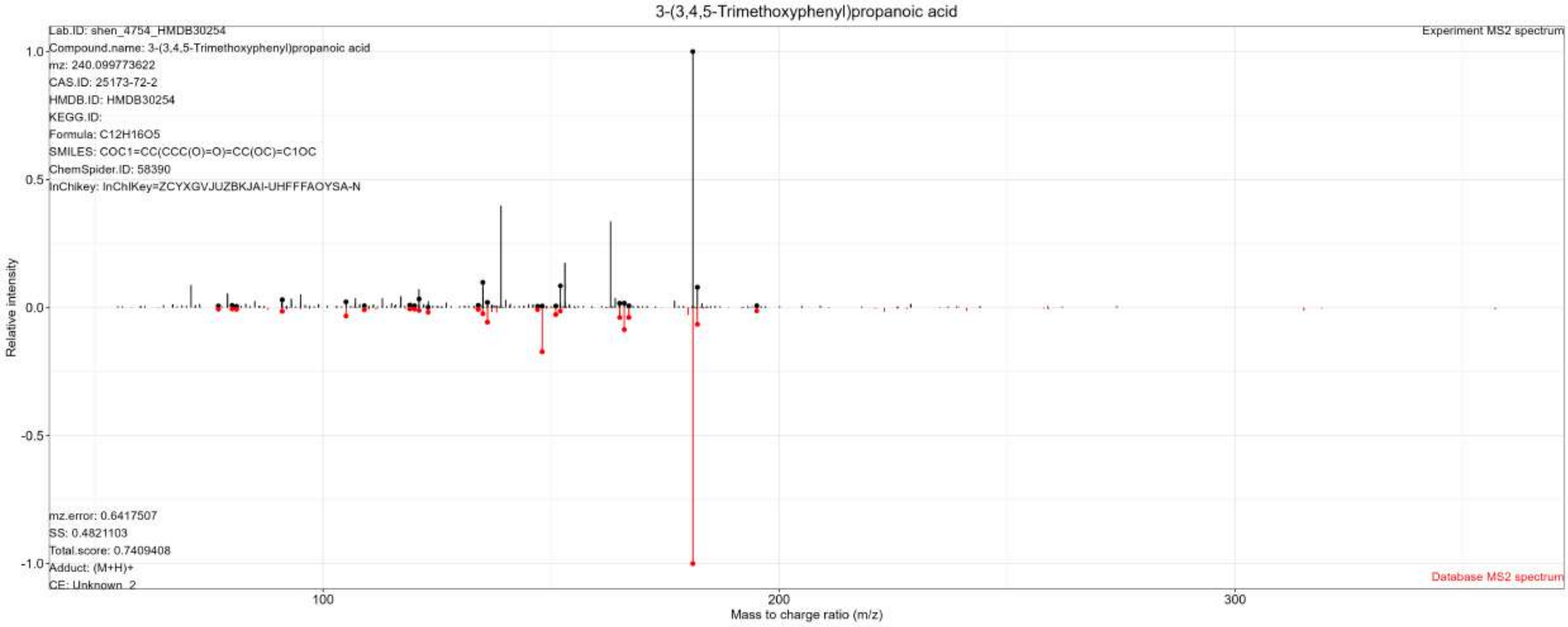
3-(3,4,5-Trimethoxyphenyl)propanoic acid
Lab.ID: shen_4754_HMDB30254
Compound.name: 3-(3,4,5-Trimethoxyphenyl)propanoic acid
mz: 240.099773622
CAS.ID: 25173-72-2
HMDB.ID: HMDB30254
KEGG.ID:
Formula: C12H16O5
SMILES: COC1=CC(CCC(O)=O)=CC(OC)=C1OC
ChemSpider.ID: 58390
InChikey: InChIKey=ZCYXGVJUZBKJAI-UHFFFAOYSA-N
Experiment MS2 spectrum
mz.error: 0.6417507
SS: 0.4821103
Total.score: 0.7409408
Adduct: (M+H)+
CE: Unknown_2
Database MS2 spectrum
Relative intensity
Mass to charge ratio (m/z)

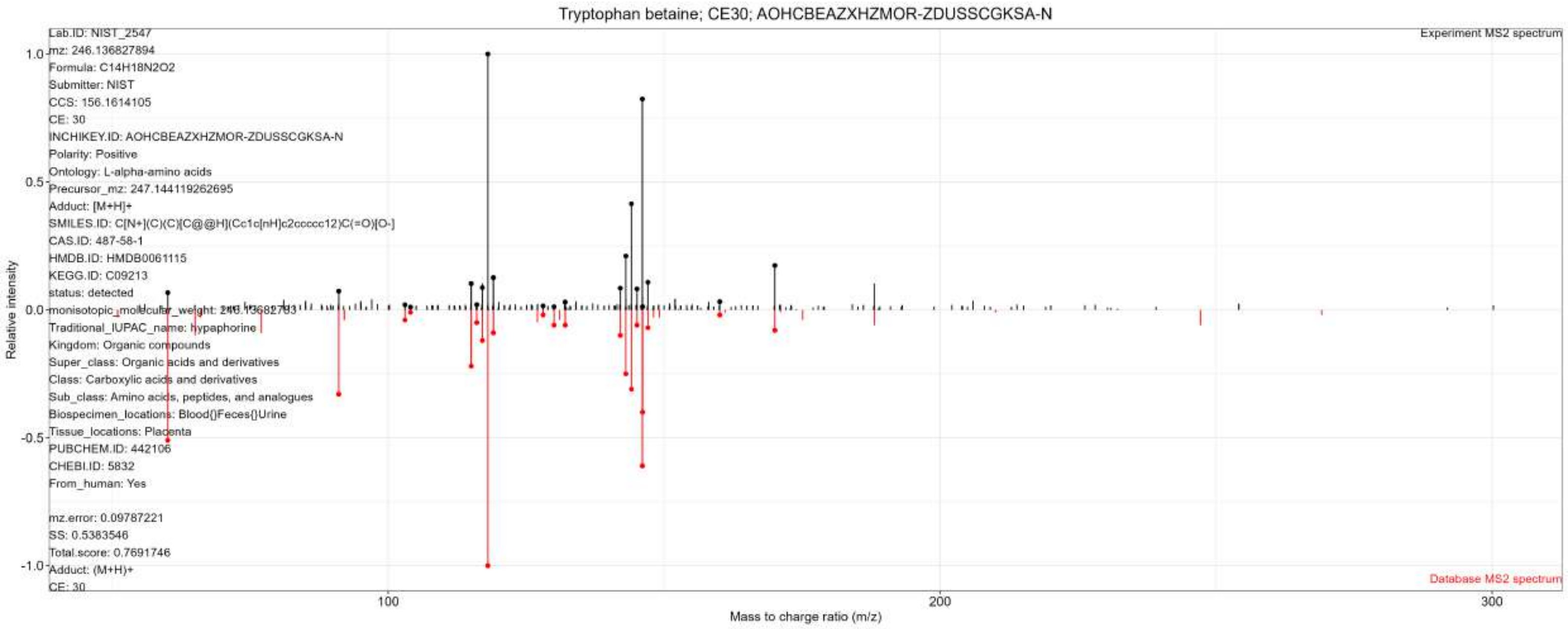
Tryptophan betaine; CE30; AOHCBEAZXHZMOR-ZDUSSCGKSA-N
Lab.ID: NIST_2547
mz: 246.136827894
Formula: C14H18N2O2
Submitter: NIST
CCS: 156.1614105
CE: 30
INCHIKEY.ID: AOHCBEAZXHZMOR-ZDUSSCGKSA-N
Polarity: Positive
Ontology: L-alpha-amino acids
Precursor_mz: 247.144119262695
Adduct: [M+H]+
SMILES.ID: C[N+](C)(C)[C@@H](Cc1c[nH]c2ccccc12)C(=O)[O-]
CAS.ID: 487-58-1
HMDB.ID: HMDB0061115
KEGG.ID: C09213
status: detected
monisotopic_molecular_weight: 246.13682783
Traditional_IUPAC_name: hypaphorine
Kingdom: Organic compounds
Super_class: Organic acids and derivatives
Class: Carboxylic acids and derivatives
Sub_class: Amino acids, peptides, and analogues
Biospecimen_locations: Blood{}Feces{}Urine
Tissue_locations: Placenta
PUBCHEM.ID: 442106
CHEBI.ID: 5832
From_human: Yes
mz.error: 0.09787221
SS: 0.5383546
Total.score: 0.7691746
Adduct: (M+H)+
CE: 30
Experiment MS2 spectrum
Database MS2 spectrum
Relative intensity
1.0
0.5
0.0
-0.5
-1.0
100
200
300
Mass to charge ratio (m/z)

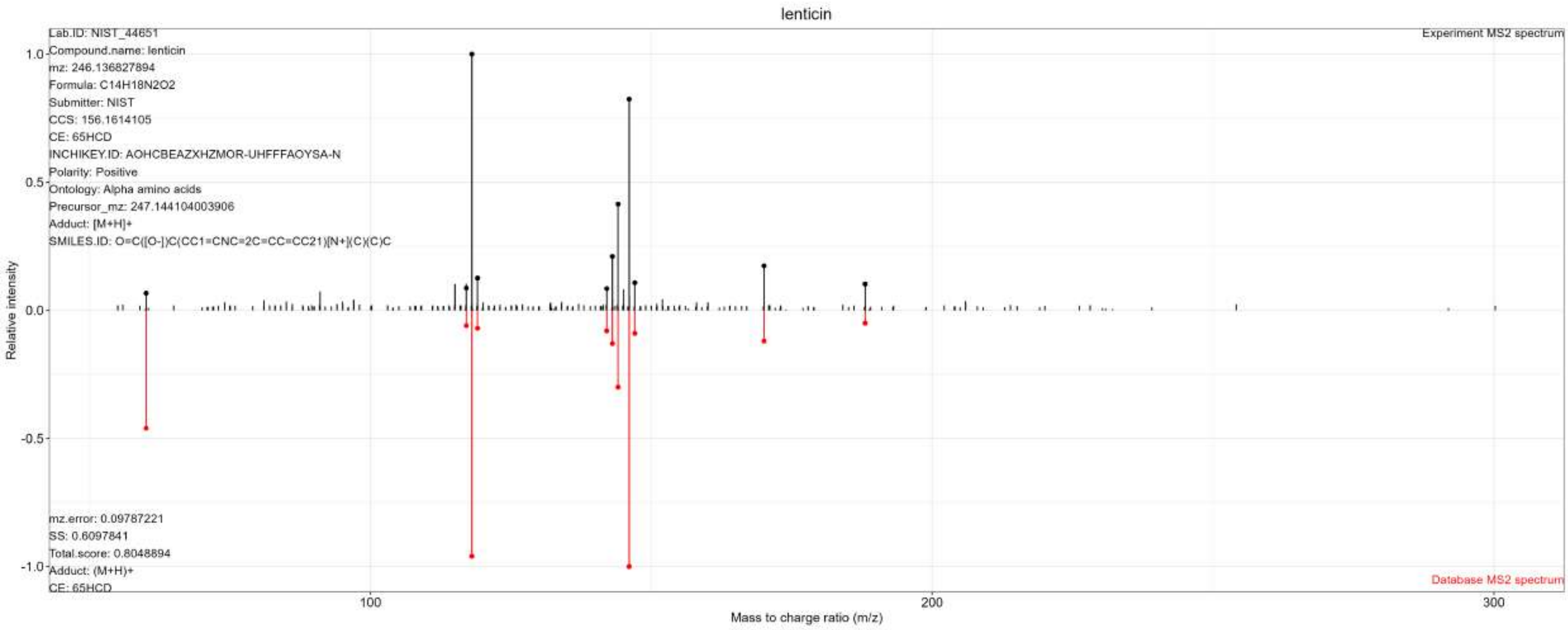
lenticin
Lab.ID: NIST_44651
Compound.name: lenticin
mz: 246.136827894
Formula: C14H18N2O2
Submitter: NIST
CCS: 156.1614105
CE: 65HCD
INCHIKEY.ID: AOHCBEAZXHZMOR-UHFFFAOYSA-N
Polarity: Positive
Ontology: Alpha amino acids
Precursor_mz: 247.144104003906
Adduct: [M+H]+
SMILES.ID: O=C([O-])C(CC1=CNC=2C=CC=CC21)[N+](C)(C)C
Experiment MS2 spectrum
mz.error: 0.09787221
SS: 0.6097841
Total.score: 0.8048894
Adduct: (M+H)+
CE: 65HCD
Database MS2 spectrum
Relative intensity
1.0
0.5
0.0
-0.5
-1.0
100
200
300
Mass to charge ratio (m/z)

Serotonin

Generic Display Report (all)

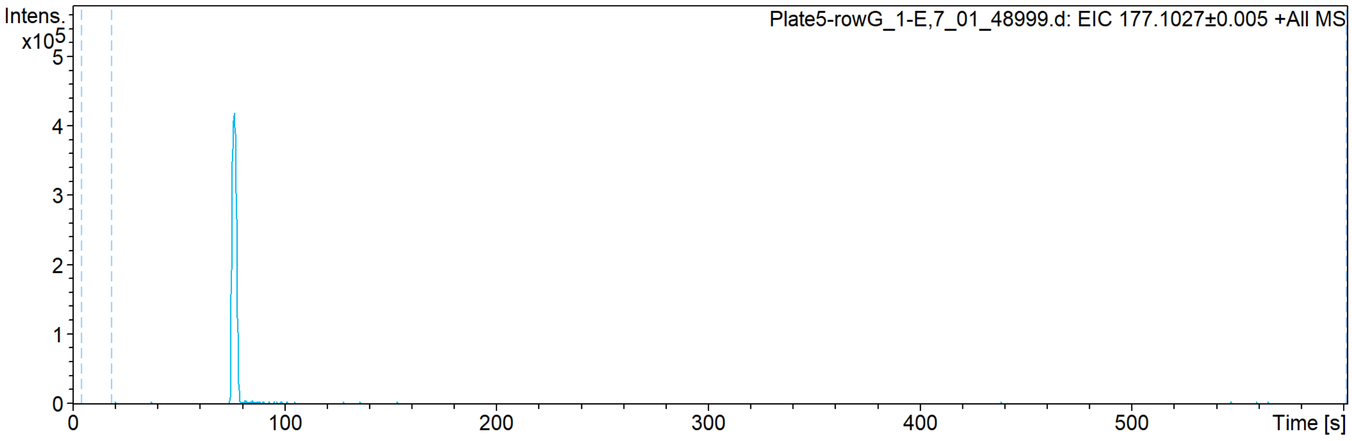

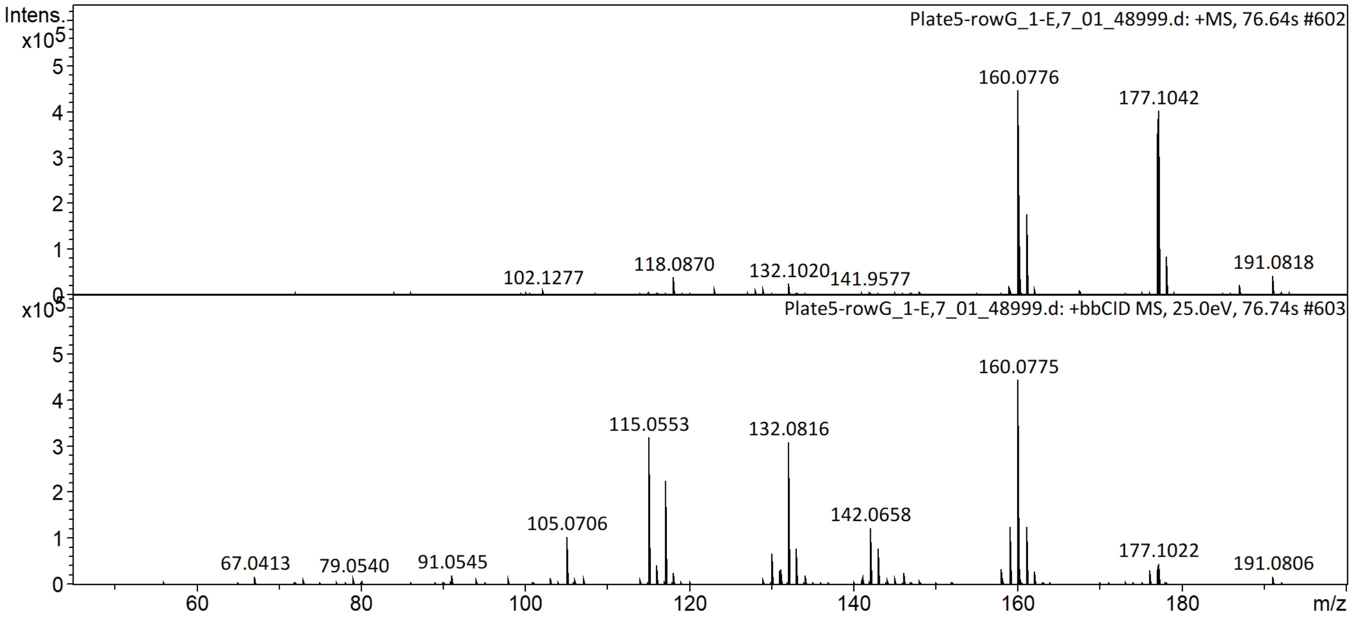

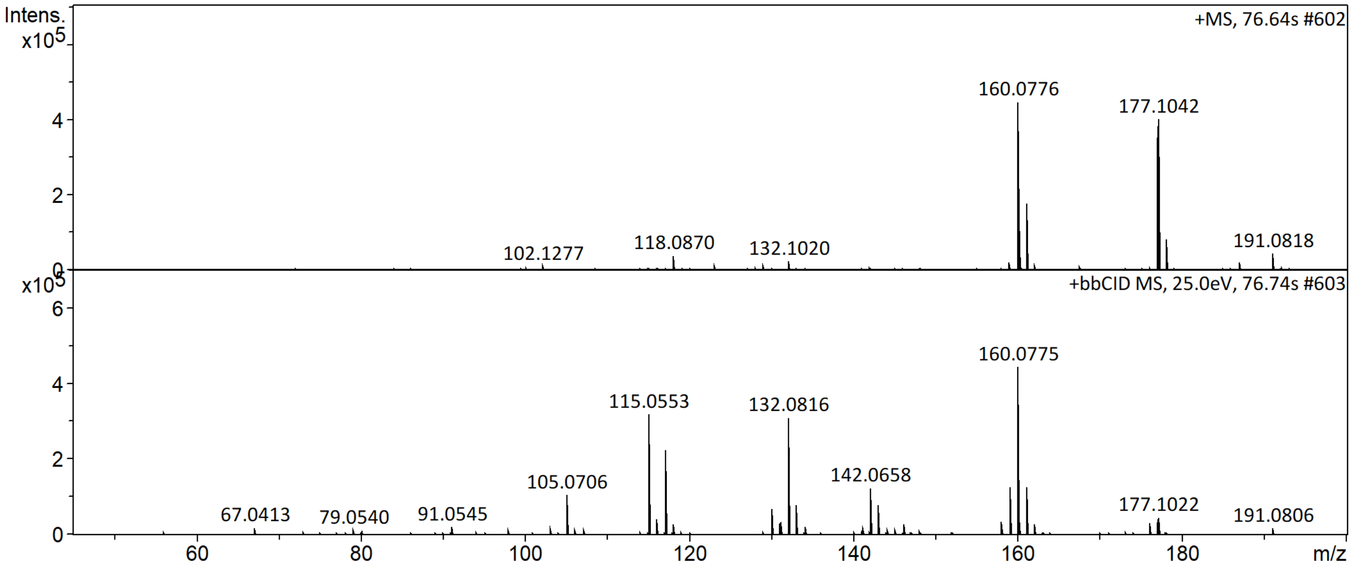

Serotonin

Generic Display Report (all)

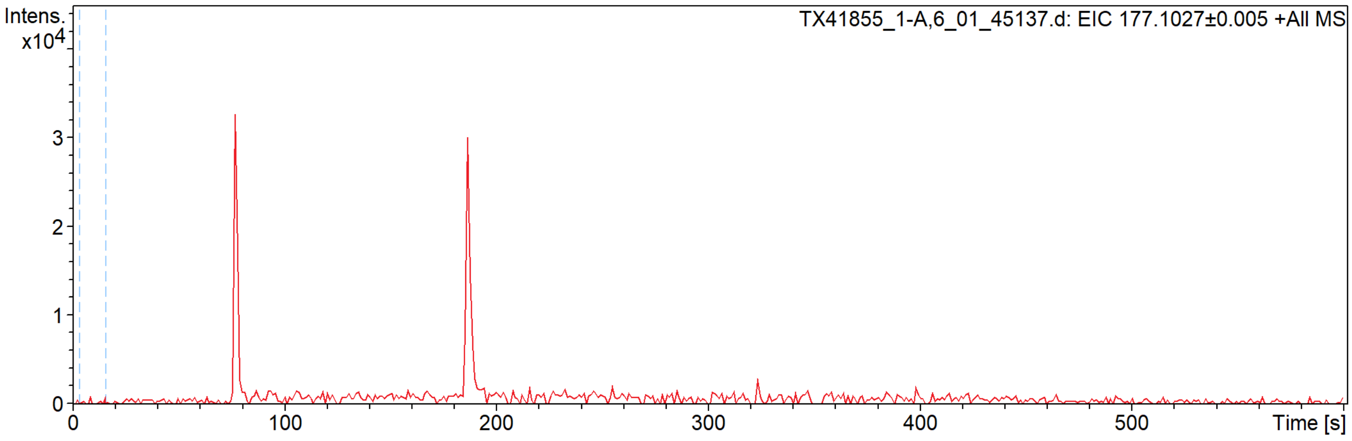

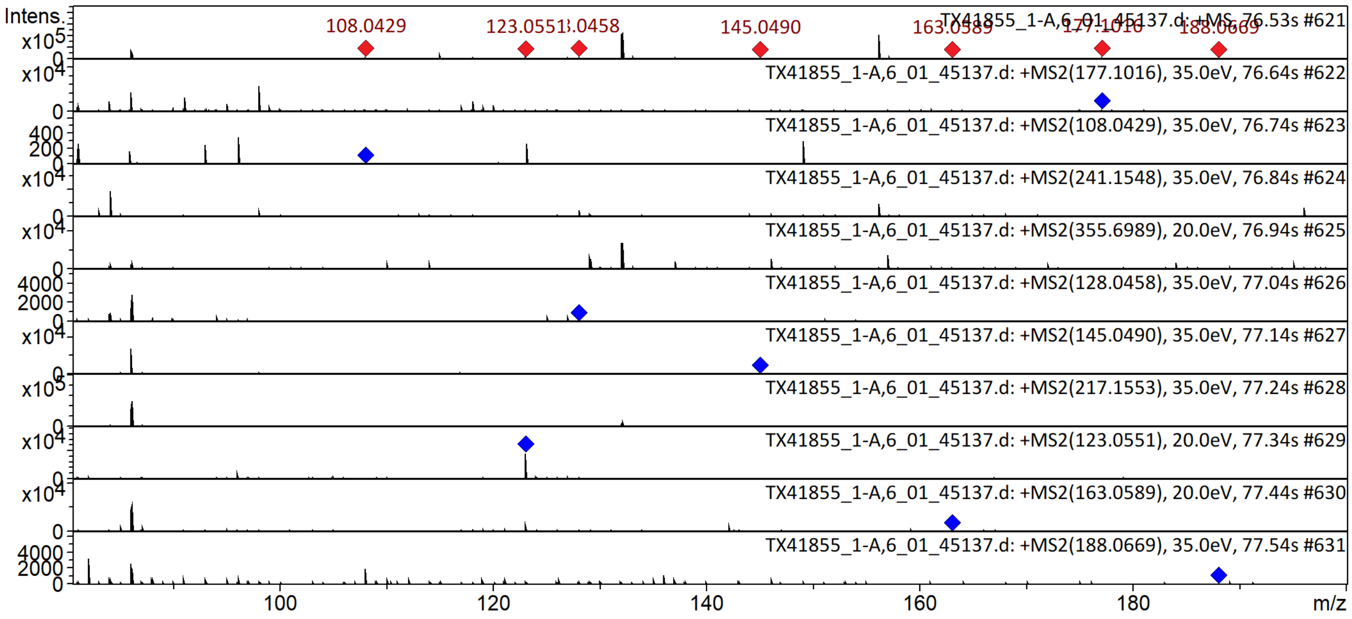

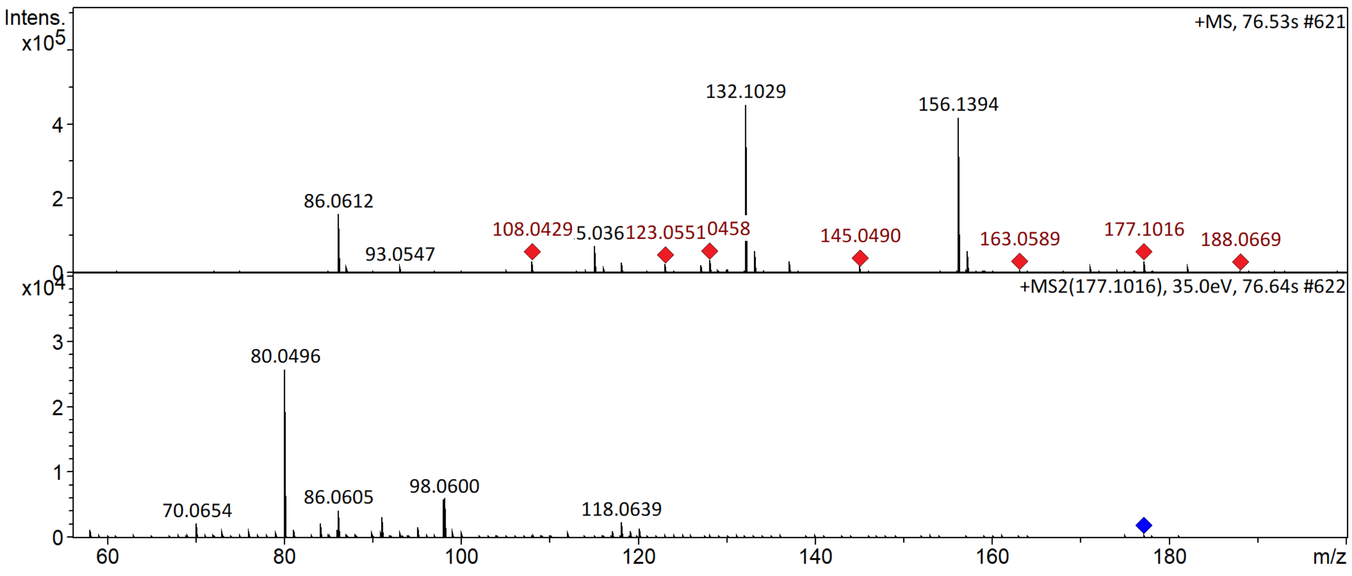

Kynurenate

Generic Display Report (all)

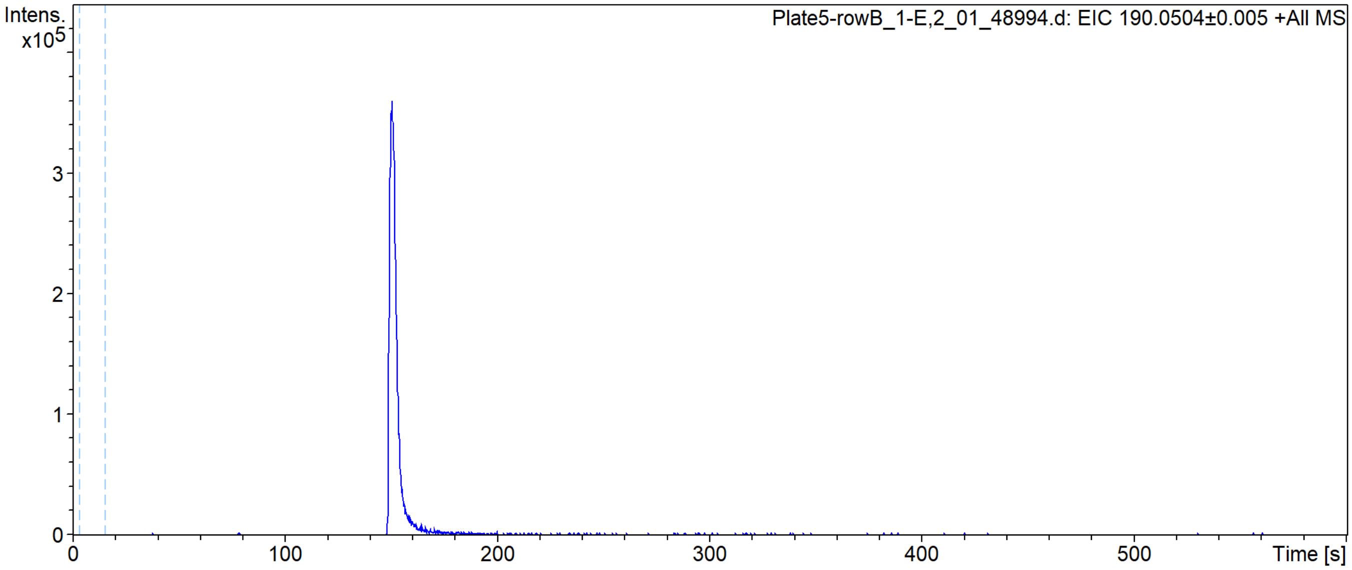

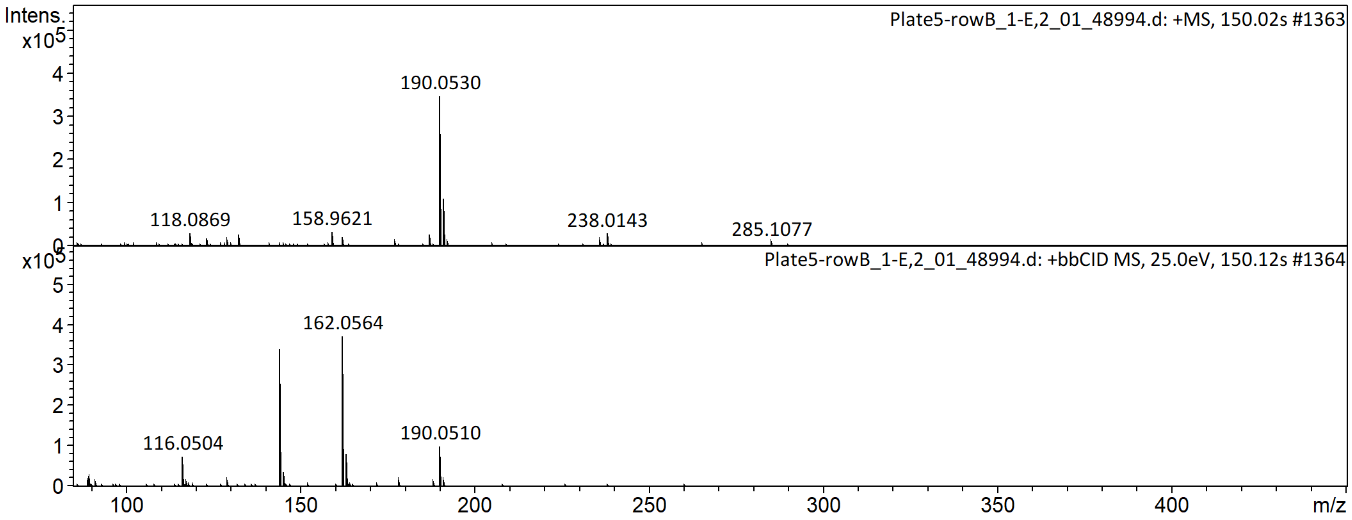

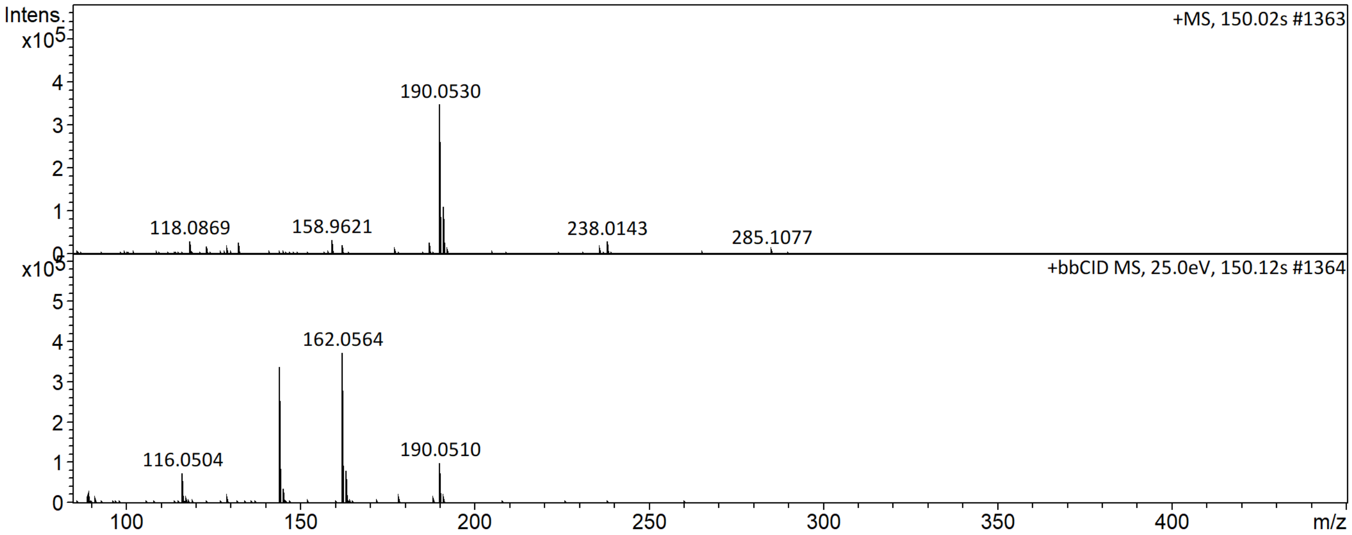

Kynurenate

Generic Display Report (all)

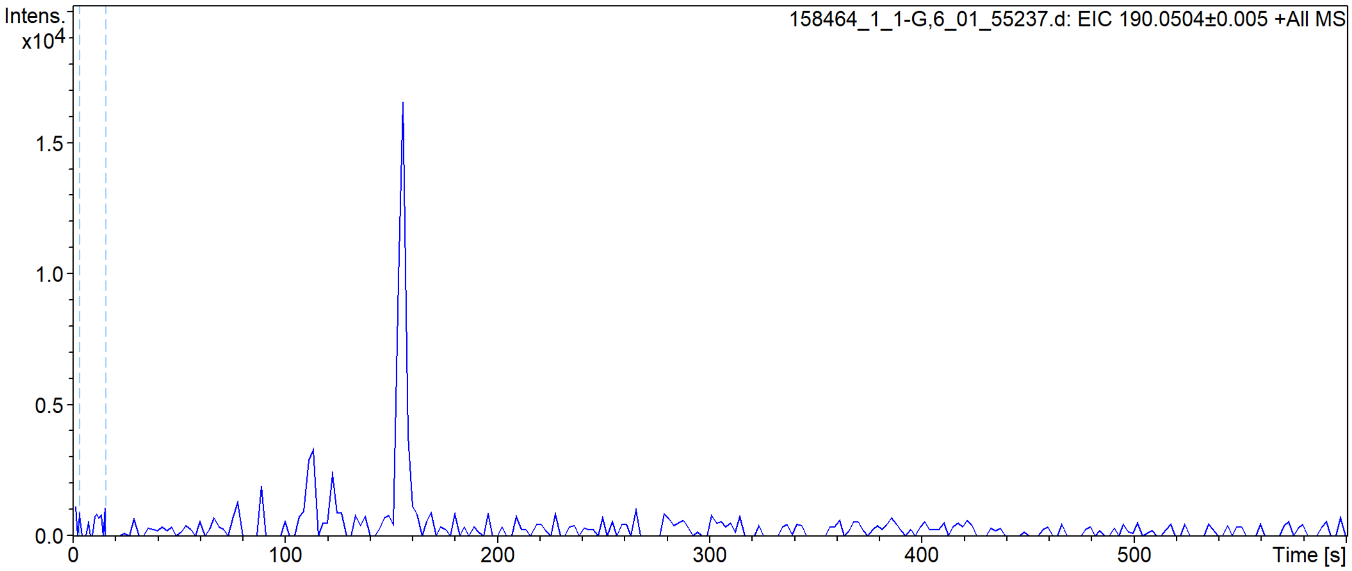

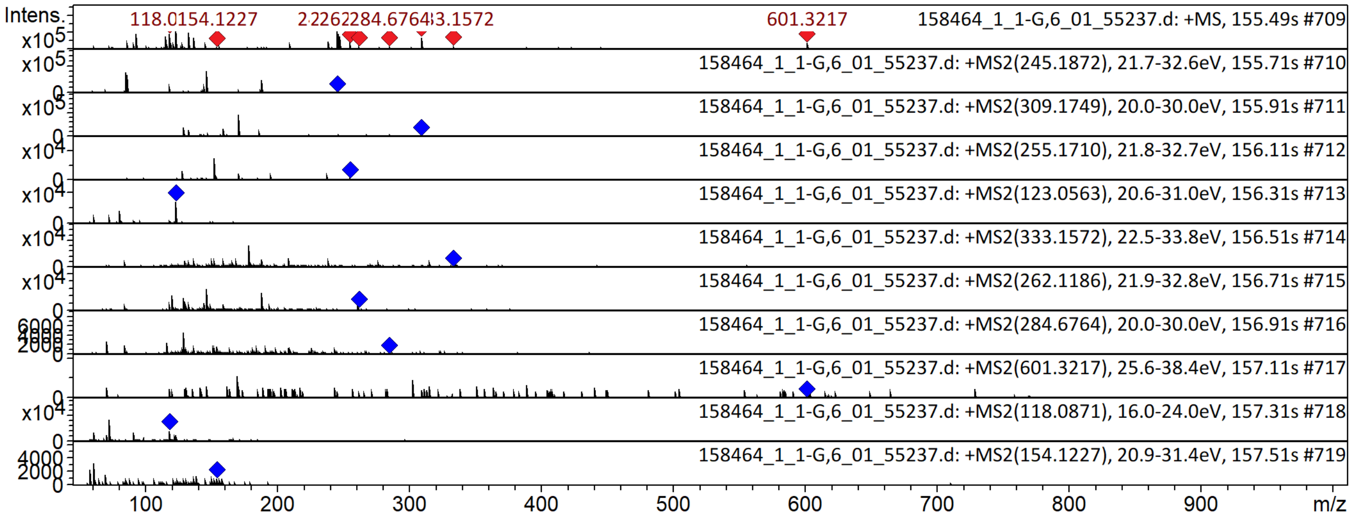

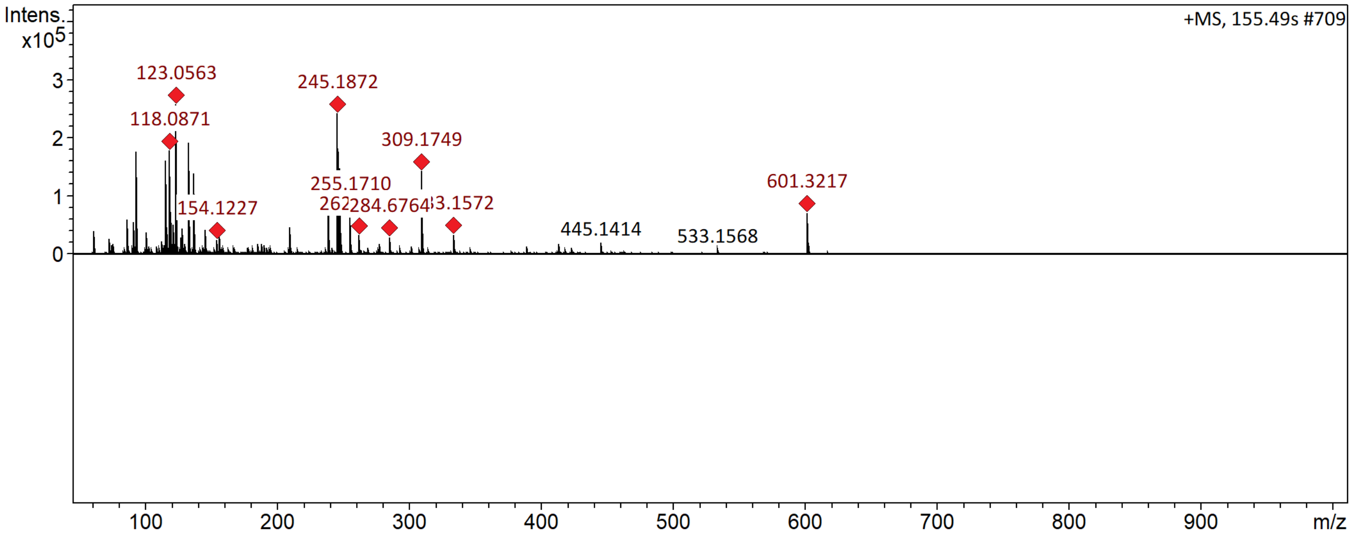

Kynurenate

Generic Display Report (all)

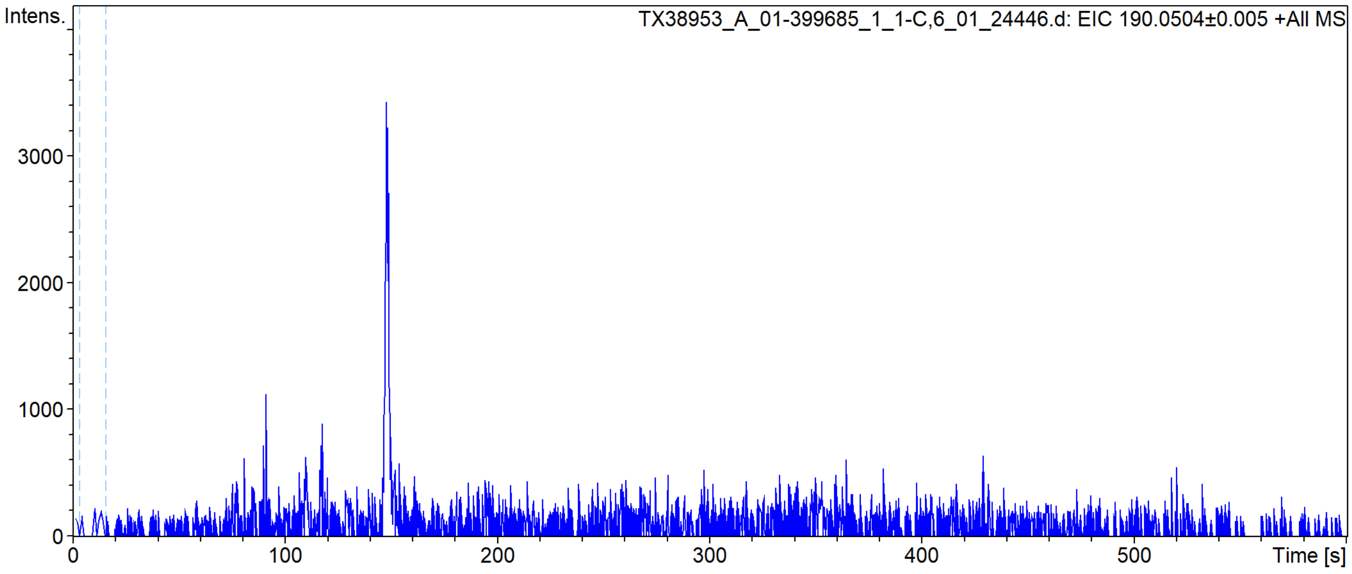

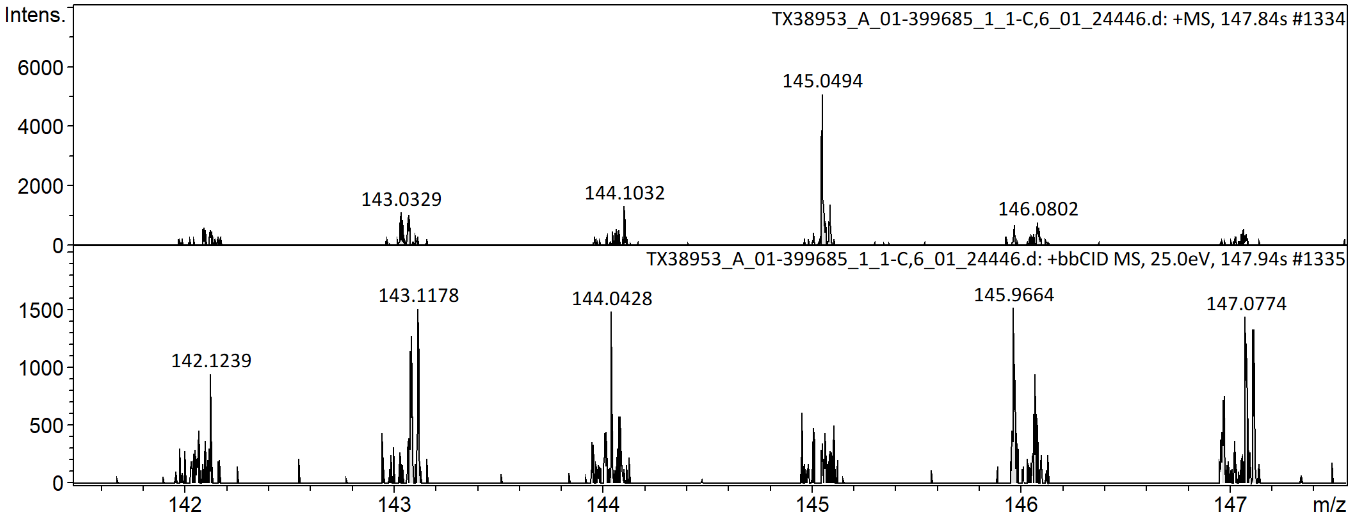

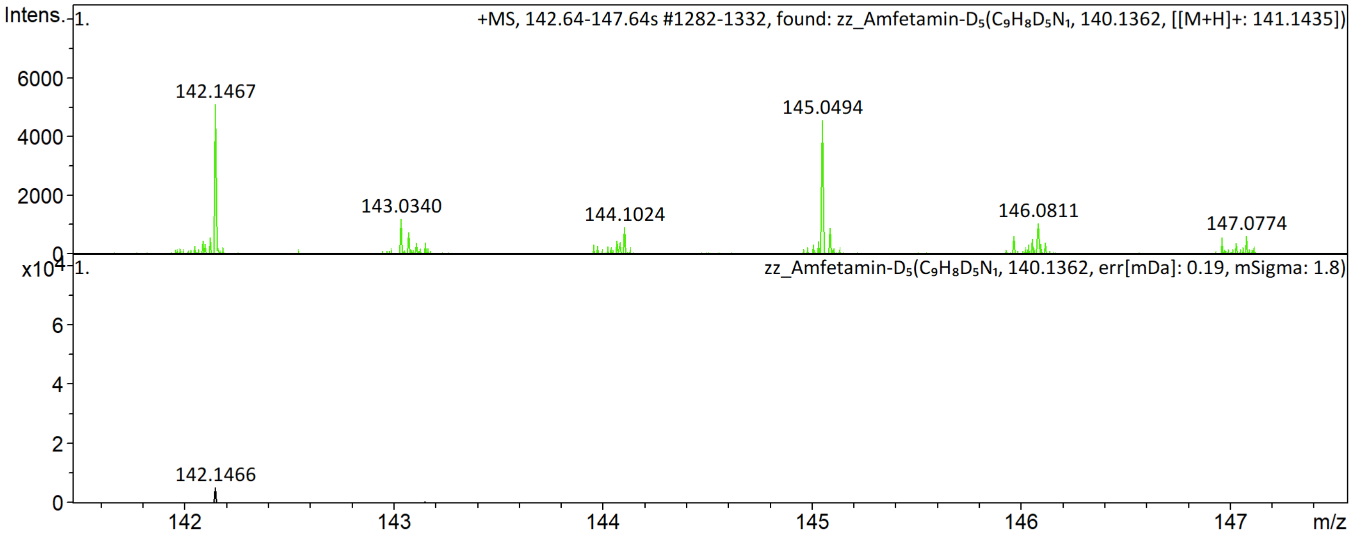

Kynurenate

Generic Display Report (all)

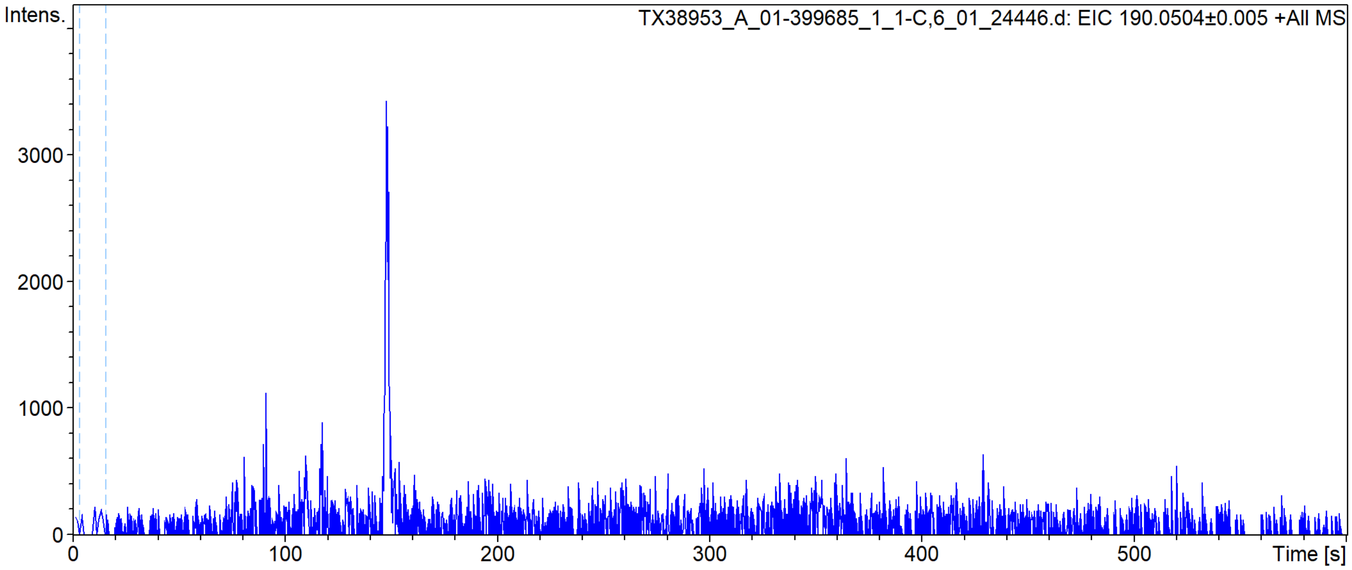

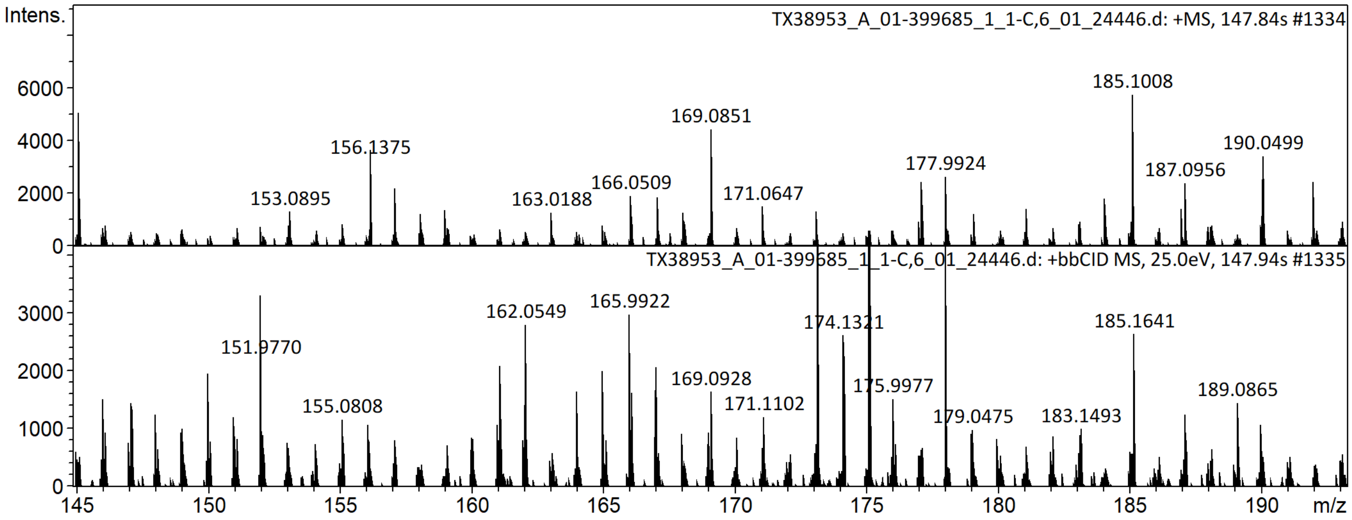

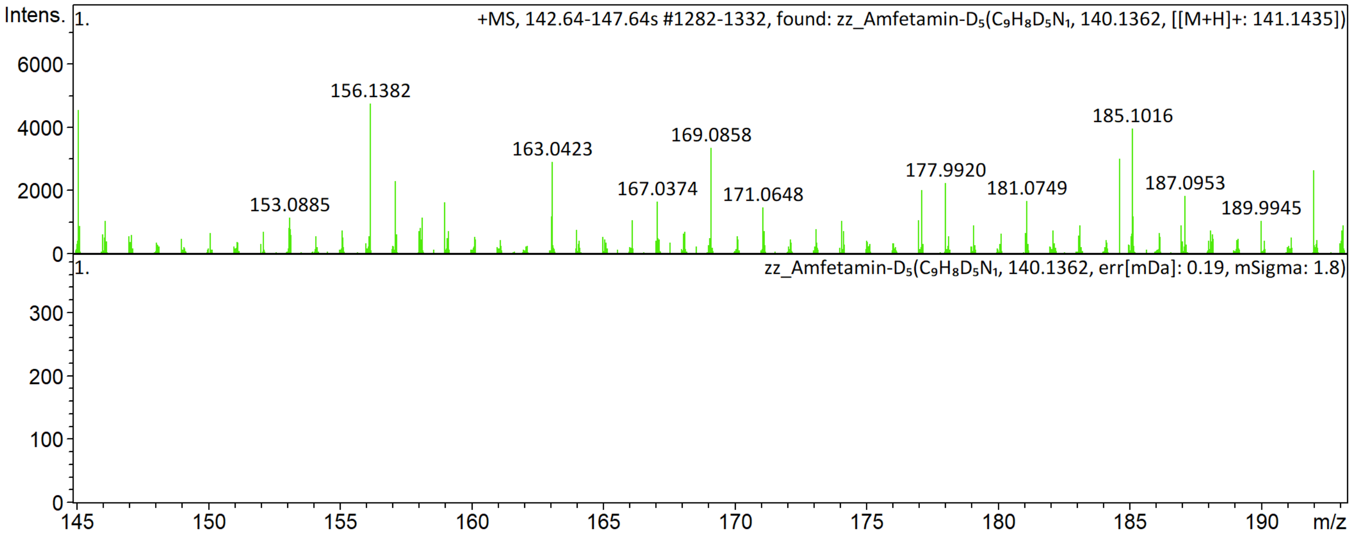

Supplement: Supplementary file 2 — Data S2: [file ACEL-22-e13813-s001.pdf]
