# Supplementary material for: Large‐Scale metabolomics: Predicting biological age using 10,133 routine untargeted LC–MS measurements
Source: Aging Cell. 2023 Mar 19;22(5):e13813. doi: 10.1111/acel.13813 (PMC10186604; doi:10.1111/acel.13813)
Supplement: Supplementary file 3 — Appendix S1. [file ACEL-22-e13813-s003.docx]

**Supplementary Information**

**Table of contents**

Figure S1
Retention times of four internal standards

Figure S2
The first 18 principal components colored by age.

Figure S3
PCA plots of data from different normalization methods.

Figure S4
Bootstrapped performance of models and normalization methods.

Figure S5
Model – normalization combinations and their predictions on the test set.

Figure S6
Most important predictors association to age.

Figure S7
Sample size allows low p-values despite weak correlations.

Figure S8
PCA on all samples using features with fdr<=0.01 from Spearman correlation.

Figure S9
The architecture of the ratio-based neural network model.

Figure S10

Ratio outputs from one of the 1000 NN models.

Table S1

XCMS peak calling: Workflow steps and parameter settings.


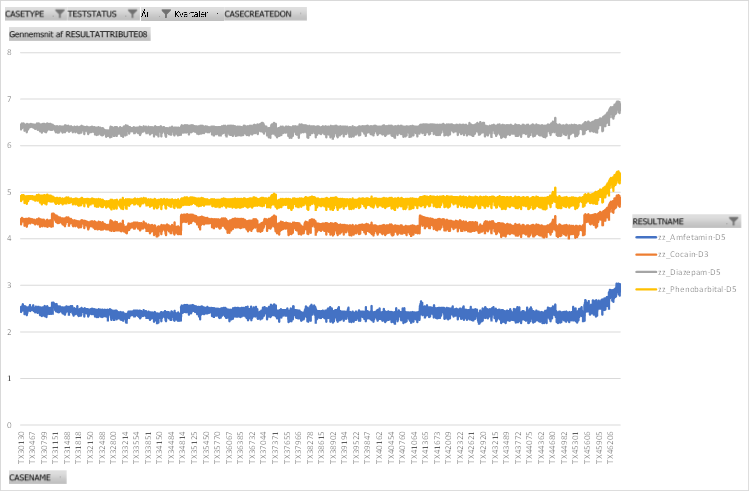


**Figure S1 | Retention times of four internal standards.** A retention time shift (y-axis) is observed every time a column is changed. Also, the third quarter of 2020 (last part of the line plot) shows a high retention time drift. The x axis is given by samples and is proportional to the sampling period from 2017 to 2020.


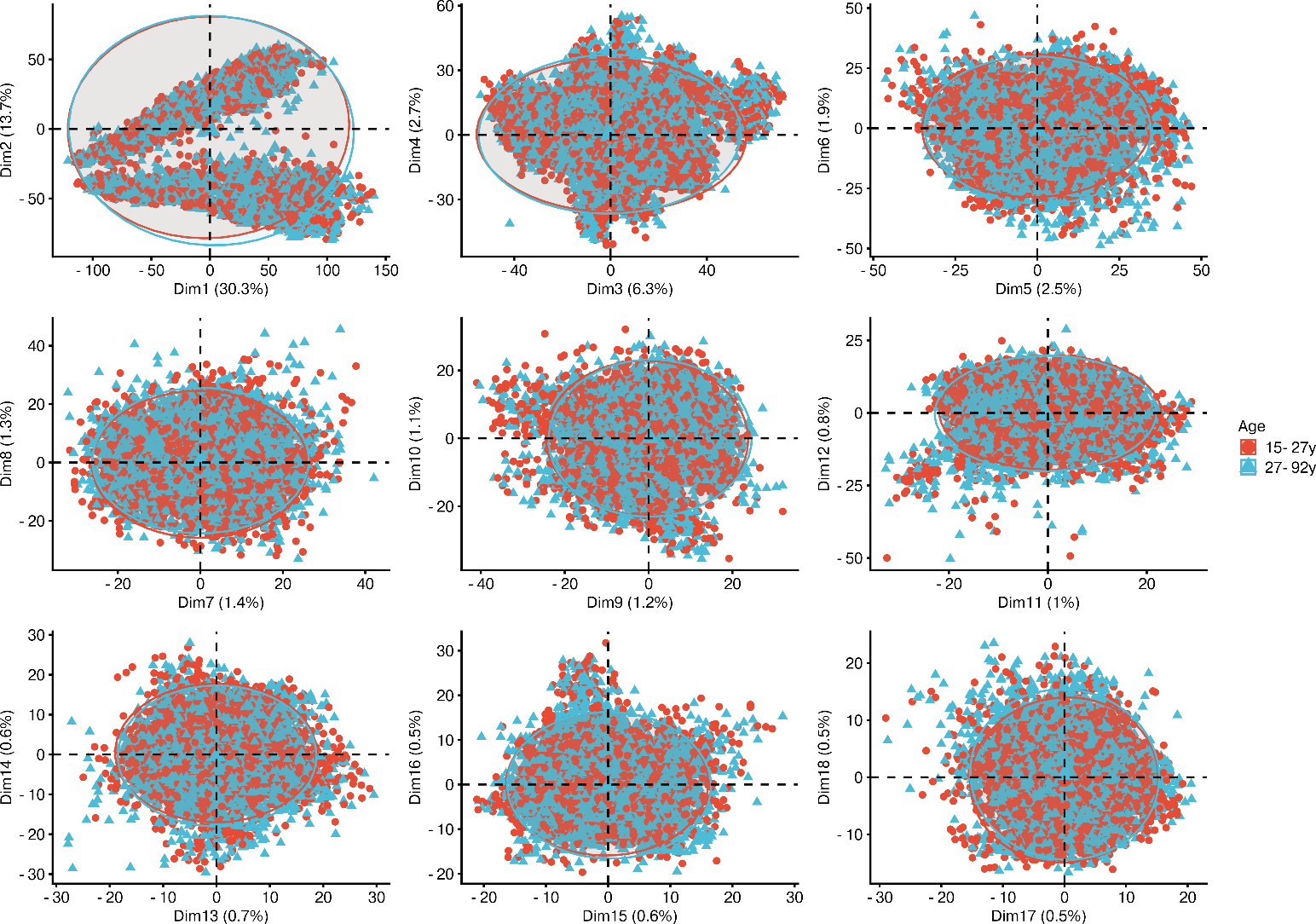
**Figure S2 | The first 18 principal components colored by age.** Colors display whether a sample is more or less than the median age. None of the components show a strong relationship to age.


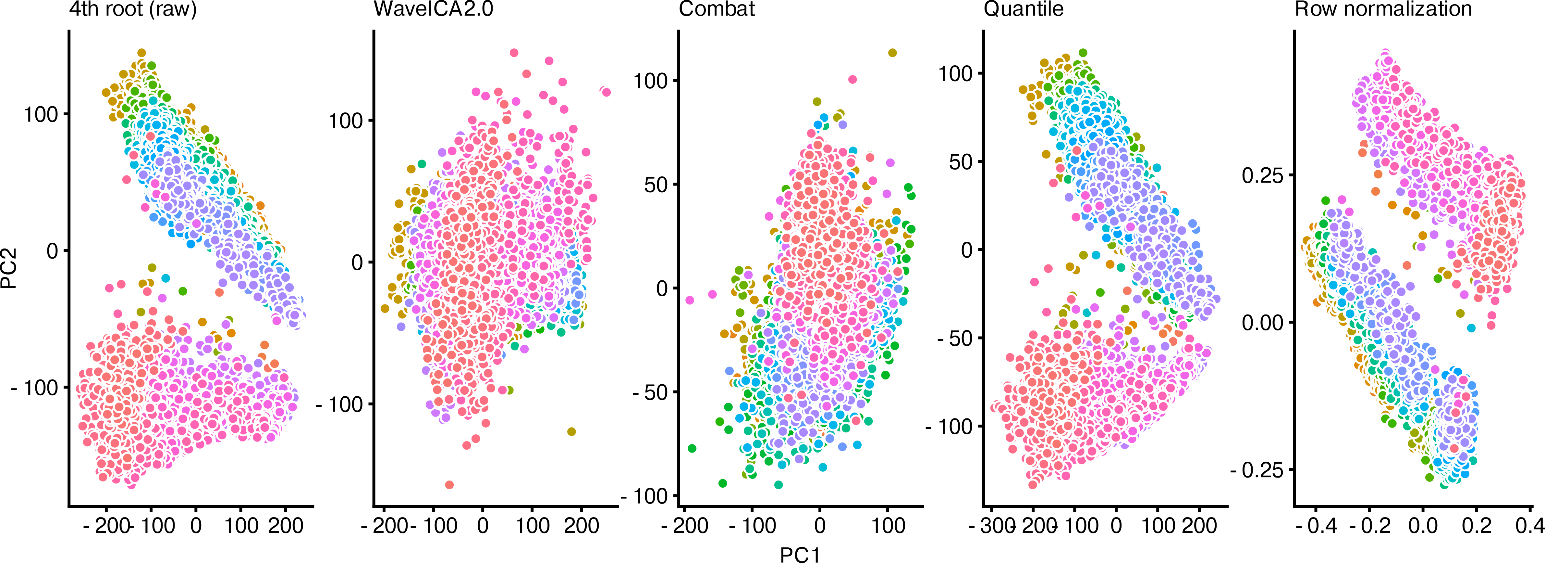
**Figure S3 | PCA plots of data from different normalization methods.** All samples are visualized, colored by batch. WaveICA2 and Combate make the batch pattern less apparent.


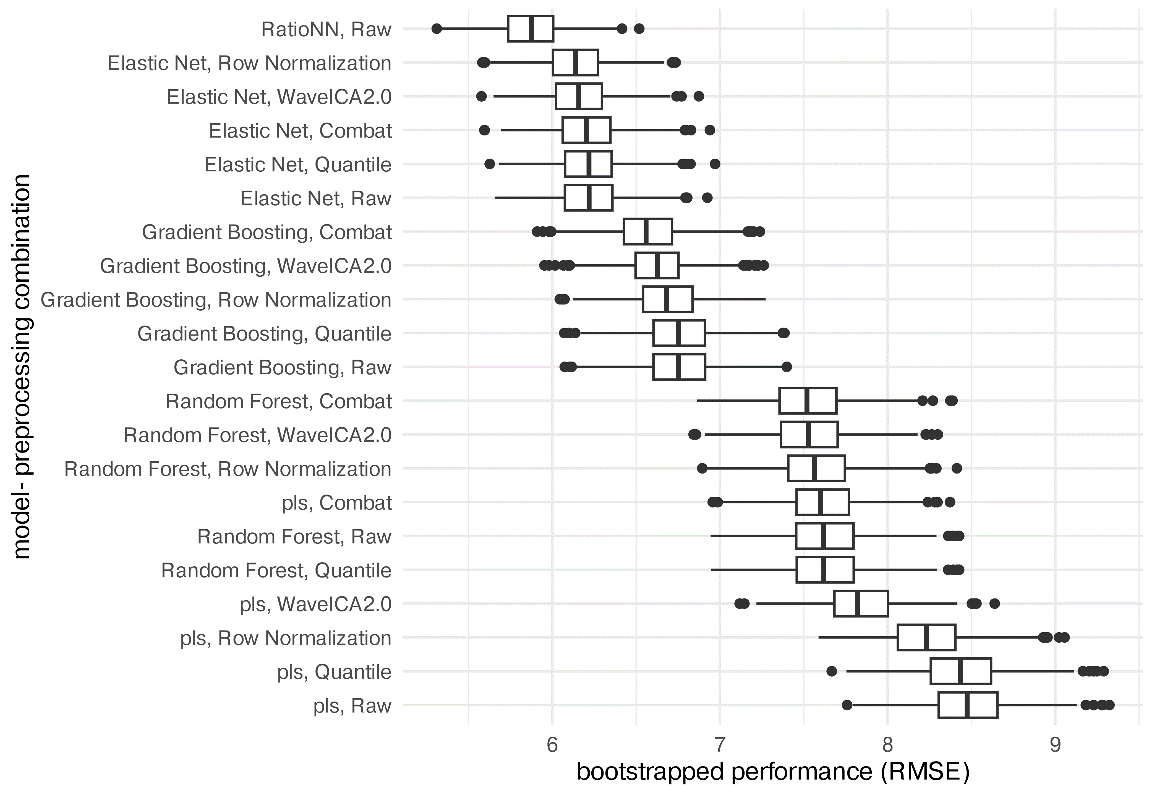

**Figure S4 | Bootstrapped performance of models and normalization methods.** The RMSE of each model was recalculated 10,000 times by resampling the predictions and observations to obtain the RMSE sampling distributions. All machine learning models had a significant effect on the performance (p-values not shown). The linear models are omitted as they have performance of +18 RMSE (table 1 in main text). Everything is based on test data (n=993).

**
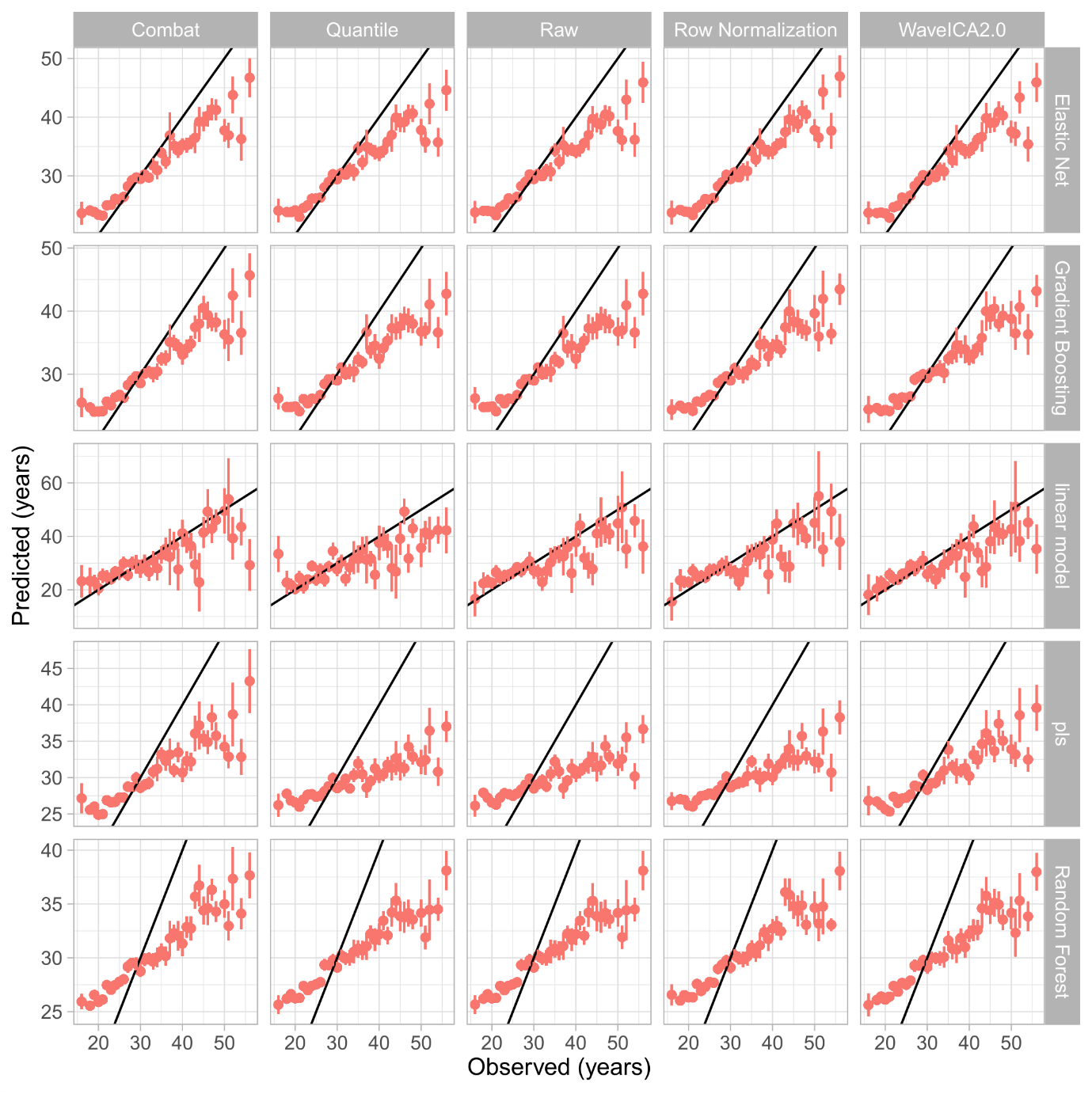
**

**Figure S5 | Model – normalization combinations and their predictions on the test set.** Performance in RMSE is reported in the main text or see figure S3.
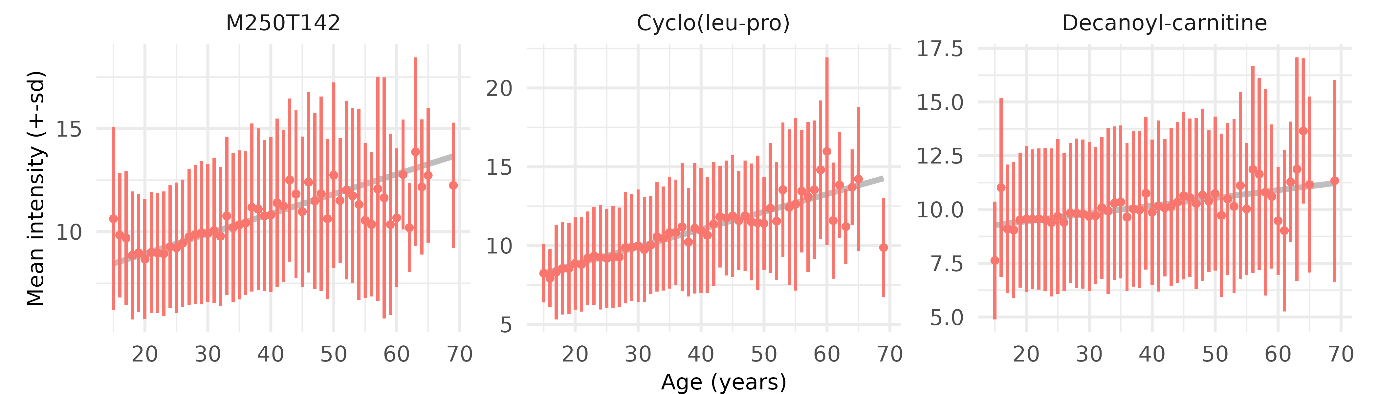


**Figure S6 | Most important predictors association to age.** The feature intensity distribution of 15-year-olds overlap with 70-year-olds.


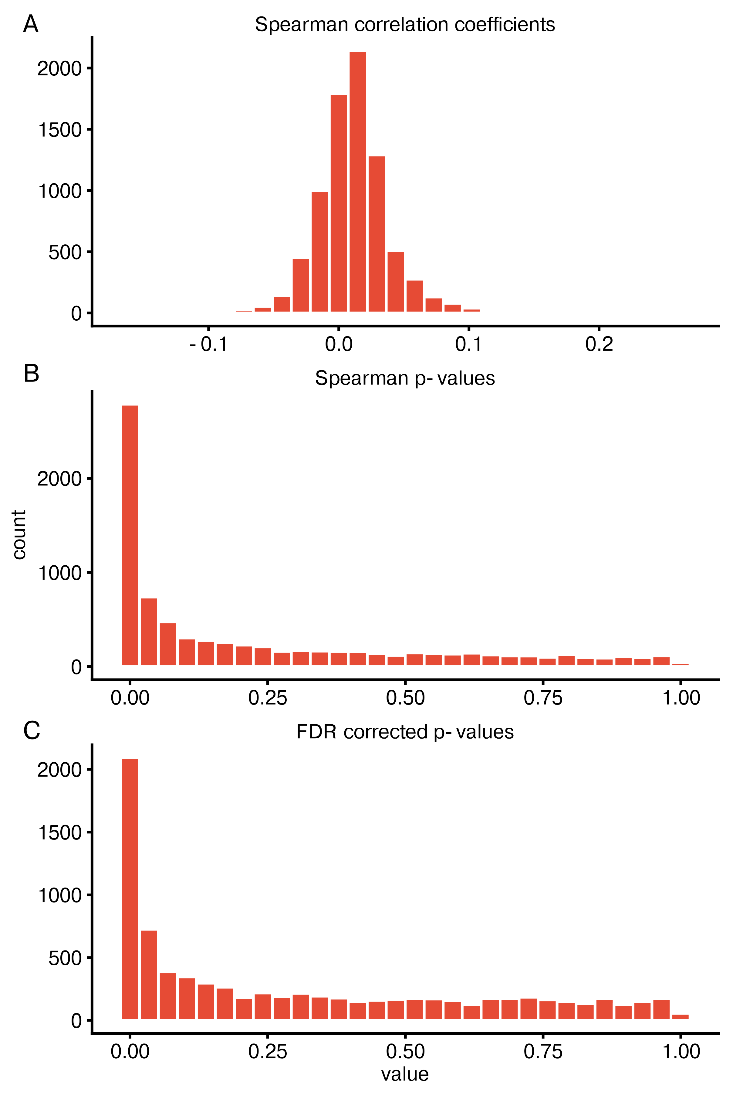


**Figure S7 | Sample size allows low p-values despite weak correlations. (**A) Distribution of spearman correlation coefficients. (B) Distribution of uncorrected *P* values from the correlation test using spearman rank correlation. (C) Distribution of FDR corrected *P*-values.


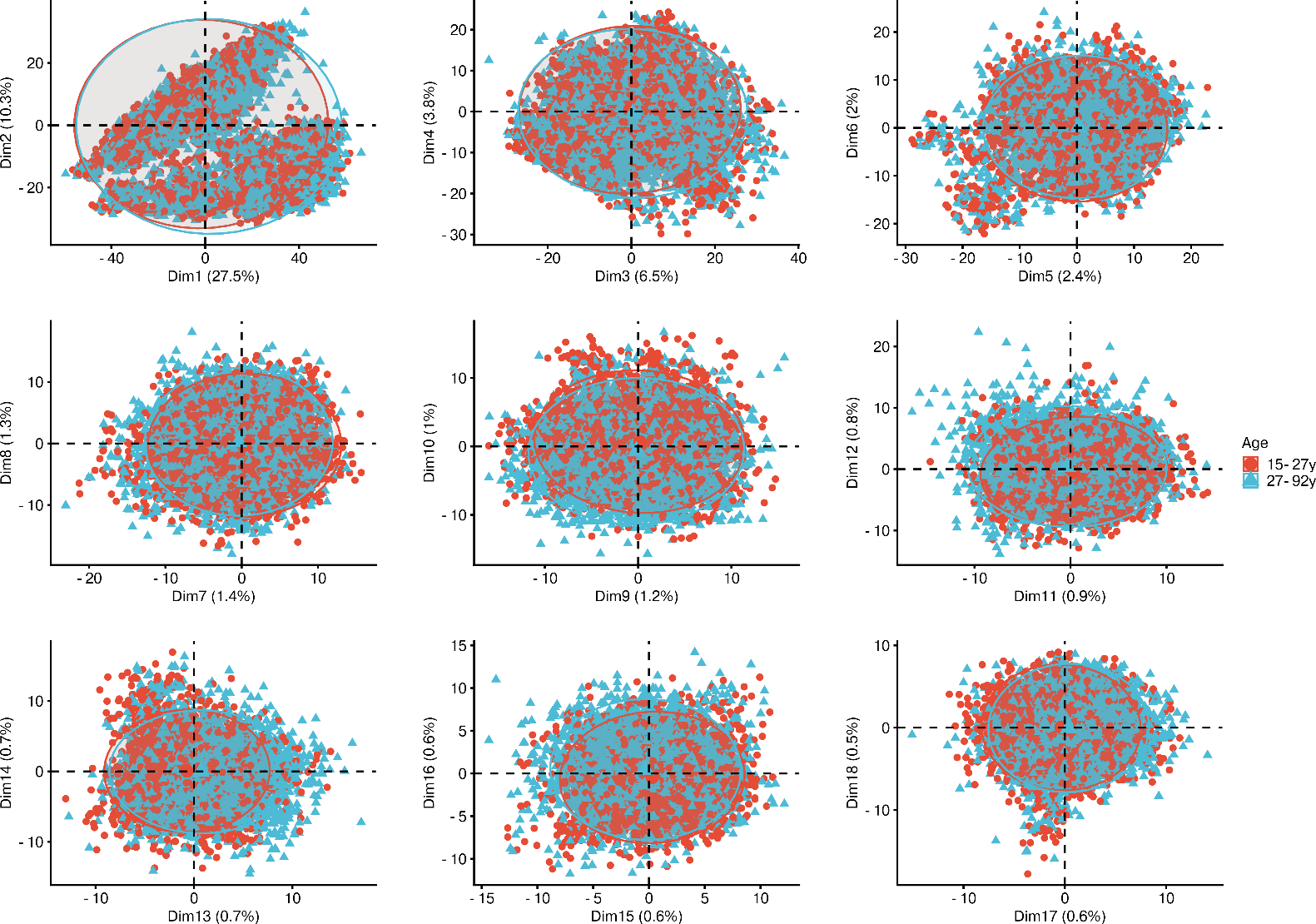
**Figure S8 | PCA on all samples using features with fdr<=0.01 from Spearman correlation.** Components 10, 13, and 16 might explain a limited amount of the age variation.


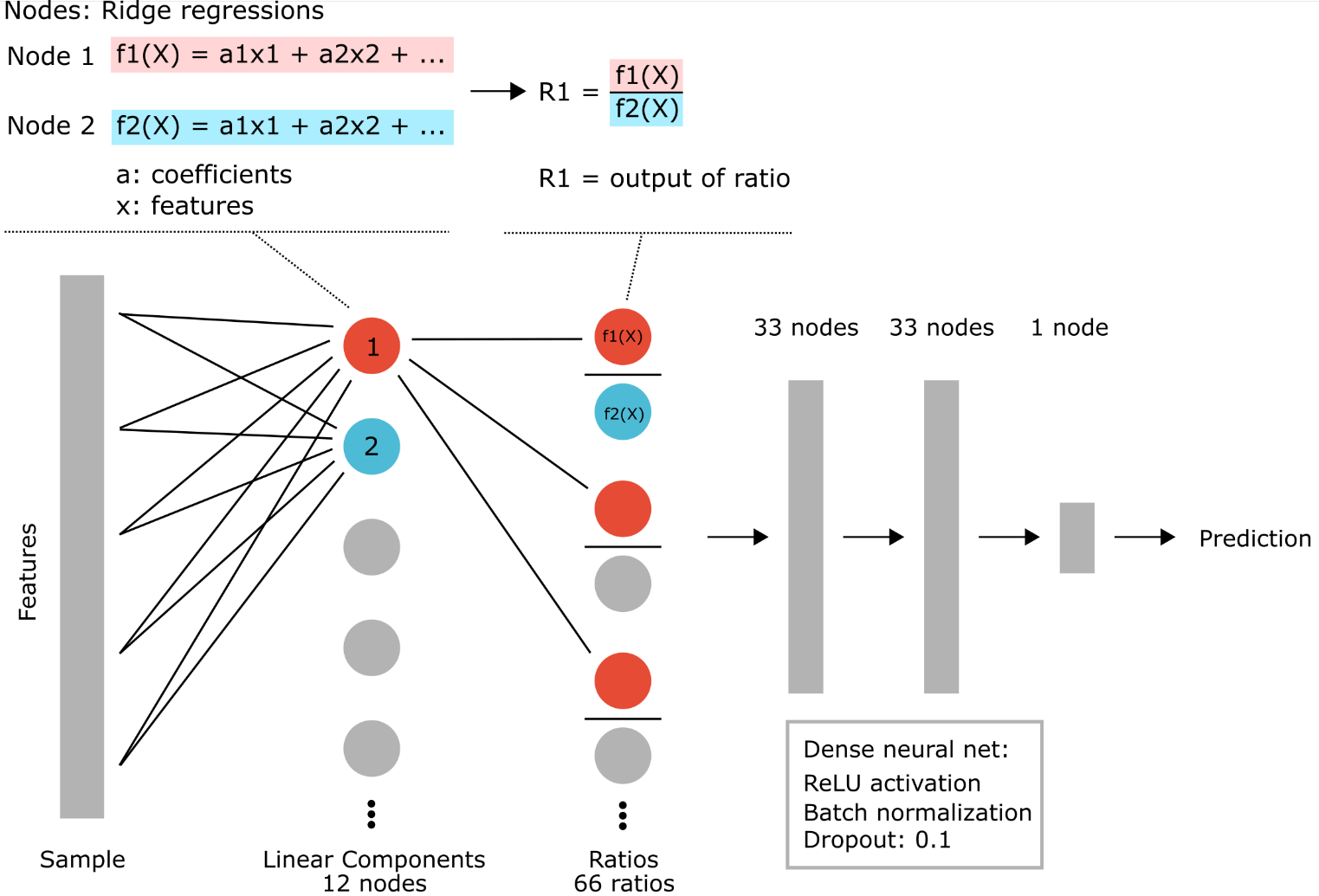


**Figure S9 | The architecture of the ratio-based neural network model.** The fourth rooted relative intensities of a sample are inputted to the first layer, which calculates the linear components. After this, ratios are calculated, batch-normalized, and transformed by the ReLU activation function. Finally, the transformed ratios are inputted to a regular Dense Neural Network (DNN).


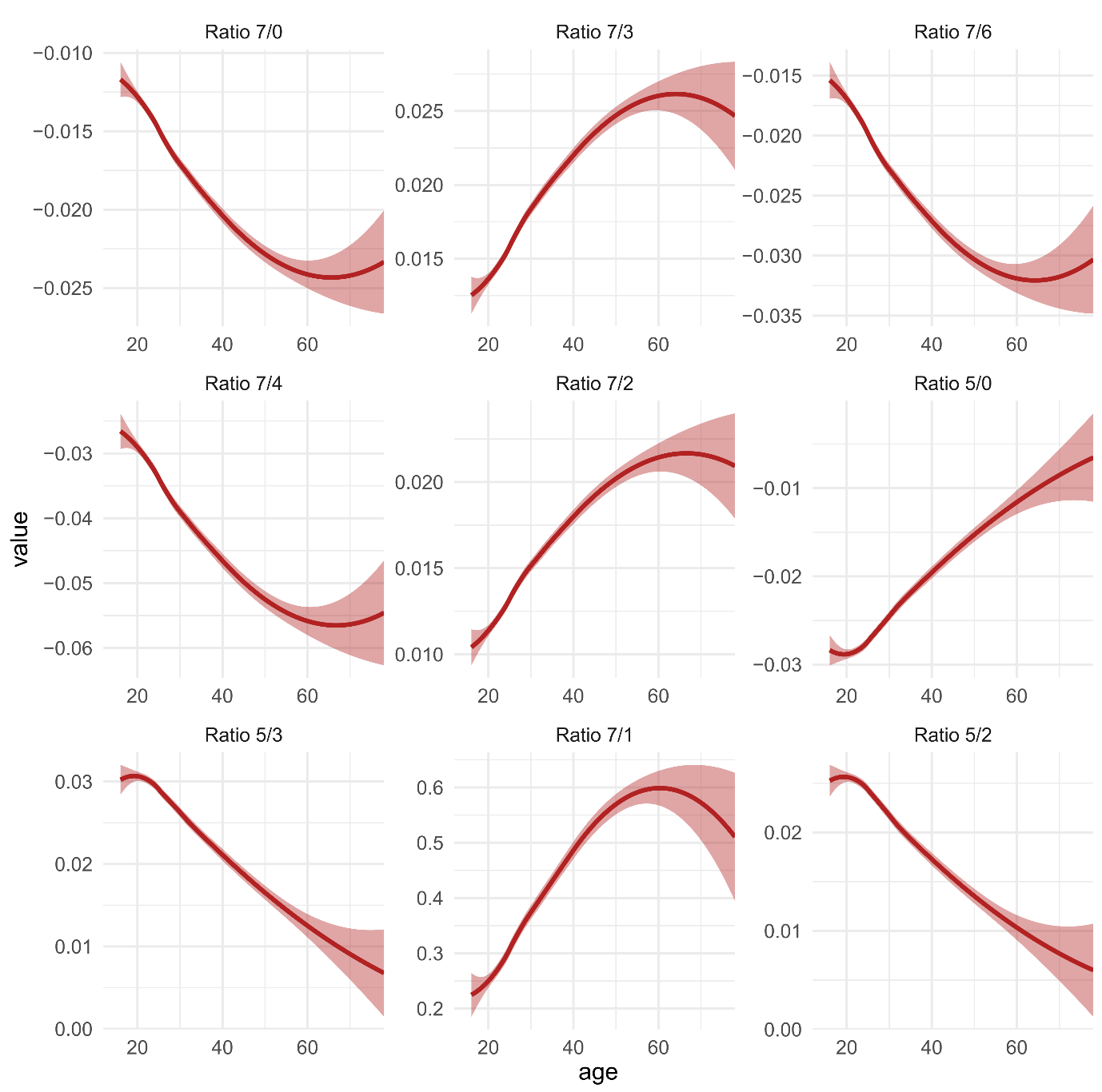


**Figure S10 | Ratio outputs from one of the 1000 NN models.** Top 20 age correlated ratios, ordered by correlation to age. The nodes (numbers) going into the ratios are outputs from the first layer in the NN model. Ratios using node 7 and node 5 are enriched. Also, nodes 0, 3, 4, and 2 seem to improve correlations.

**Table S1** | XCMS peak calling: Workflow steps and parameter settings.

| XCMS step  *(algorithm)* | Parallelized (file-wise) | Parameters (as described in R) |
| --- | --- | --- |
| Peak detection  *(centwave)* | Yes | Peakwidth: (4, 30)  snthresh: 6  ppm: 12  prefilter: (3,200)  mzdiff: 0.01 |
| Peak grouping 1  *(density)* | No | Bw: 10  minFraction: 0.5  sampleGroups: month and year of sampling |
| Peak alignment 1  *(peak groups)* | No | Smooth: loess  minFraction: 0.5  span: 0.25  extraPeaks: 250 (high due to sample size)  family: gaussian |
| Peak grouping 2  *(density)* | No | bw: 2.5  minFraction: 0.5 |
| Peak alignment 2  *(peak groups)* | No | Smooth: loess  minFraction: 0.5  span: 0.25  extraPeaks: 20  family: gaussian |
| Peak grouping 2  *(density)* | No | bw: 2.5  minFraction: 0.5 |
| Peak Integration - missing values | Yes | Default parameters |
